# Experimental and numerical investigations of double bracket to CHS column joints

**DOI:** 10.1038/s41598-024-77646-6

**Published:** 2024-11-07

**Authors:** Ahmed H. Abdelaal, Tamer H. Radwan, Amr A. Shaat

**Affiliations:** Department of Structural Engineering, Ain Shams University, Cairo, Egypt

**Keywords:** Bracket, CHS, Column, Joint, Tension, Eccentricity, Engineering, Civil engineering

## Abstract

The primary objective of this study is to precisely characterize the behavior of double bracket-to-circular Hollow Section (CHS) column joints due to combined internal forces resulting from double tensile loading in opposite directions. In order to accomplish this goal, an experimental program consisting of eight test specimens has been carried out and numerical finite element modeling has been employed for the same specimens to analyze the stresses and deformations that occur within the vicinity of bracket-to-CHS joints. A total of 15 finite element models were constructed to simulate the initial study, addressing boundary conditions and facilitating verification and results comparison. The study included an investigation of various parameters, including the spacing between brackets in the longitudinal direction, as well as the depth-to-thickness ratio of the CHS columns and adding a T-stiffener to the bracket configuration. The study determined that an increase in the diameter-to-thickness ratio of the CHS columns significantly reduced the overall strength of the joint. Furthermore, findings suggested that increasing the longitudinal spacing between brackets resulted in an increase in single-bracket joint strength and a minor reduction regarding joint strength considering the effect of line loading. Moreover, adding a T-stiffener shape for the brackets enhanced the joint strength and prevented bracket tip fracture. In addition, a distinct behavior arises when considering joints with positive eccentricity, where the forces’ line of action extends beyond the circular cross-section of the CHS. In such cases, a reduction in joint strength is observed. Finally, a modification factor “A” is applied to the X-type branch plate-to-CHS strength equation presented by the AISC360-22 to account for the longitudinal spacing between brackets.

## Introduction

CHS members have been frequently employed in various structures, including iconic landmarks. These structures often feature tensile membrane systems, which are characterized by their ability to span large areas with an appealing aesthetic presence. Structural integrity between fabric membrane prestressing forces and shape form must be carefully considered. The Fabric membrane stability hinges on the application of prestressing forces across the membrane surface. The magnitude of the prestressing forces is calculated to achieve the desired membrane curvature. These forces may slightly adjust their direction in response to significant fabric deformations resulting from factors such as wind loading. This critical phase accommodates the complexities of nonlinear fabric behavior, coping with substantial displacements, and membrane actions to resist loads.

Textile fabric membranes are typically affixed to Circular Hollow steel Sections (CHS) using prestressed cables, small reeling pipes and/or welded steel brackets. Multiple brackets are employed to support membrane tensile forces providing forces equilibrium. The membrane forces which are necessary for membrane stability are transmitted to the supporting steel structure using these brackets.

**Fig. 1:**
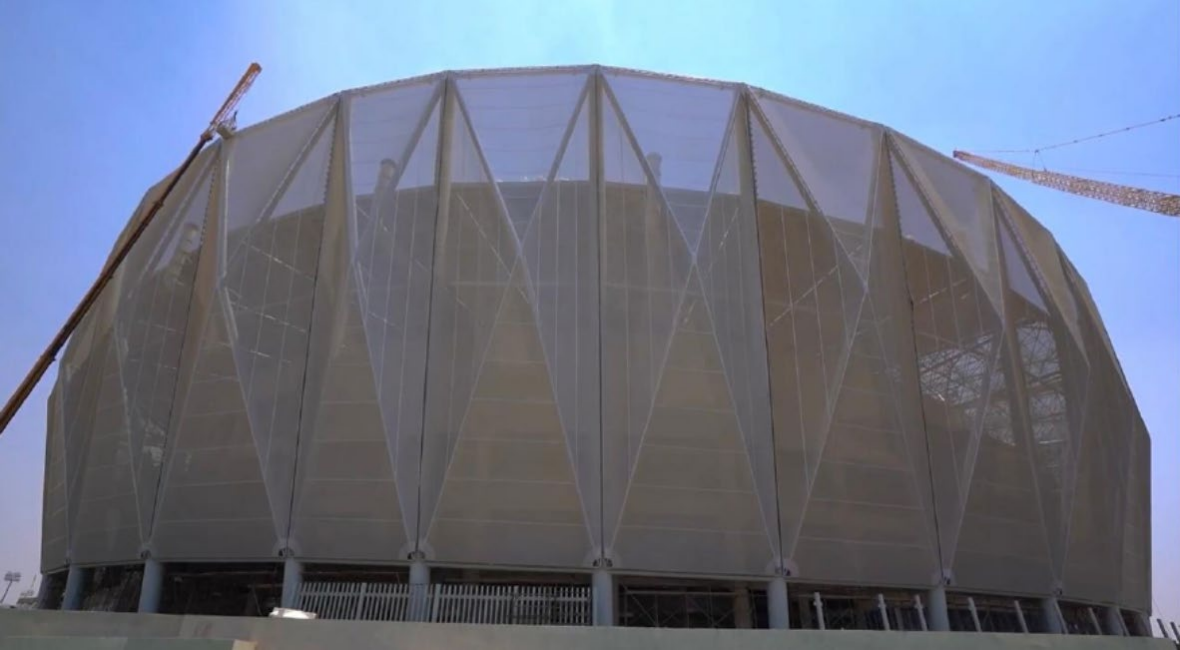
The Olympic stadium in the new administration capital, Egypt. (Taken by Dr.Tamer H.Radwan).

**Fig. 2:**
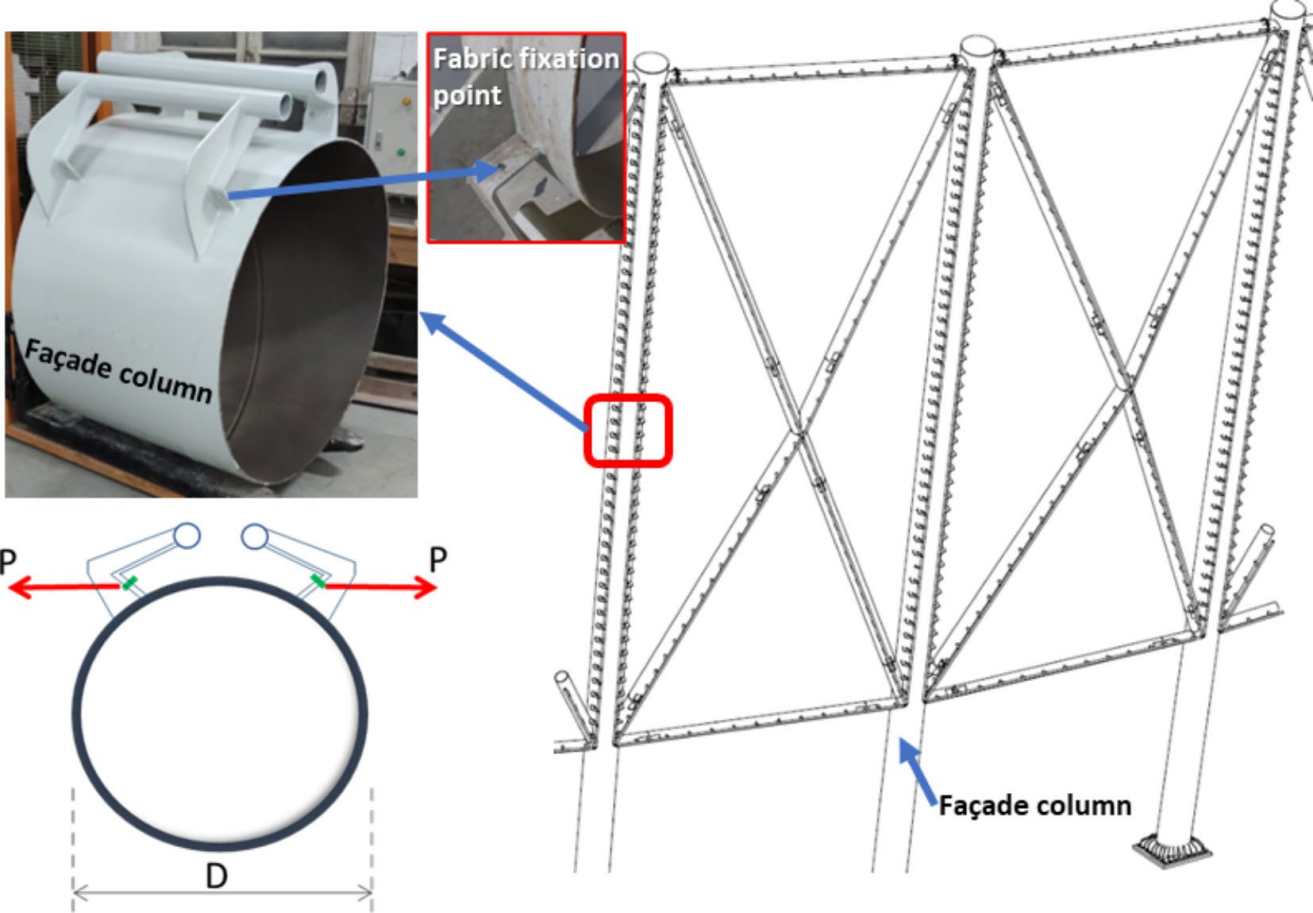
Textile membrane fabric joint to CHS supporting column.

The newly constructed Olympic stadium in Egypt’s new administrative capital introduces a catching outer façade with modern tensile fabric membranes, as shown in Fig. 1. This research is significantly motivated by the façade membrane structure of this stadium. The façade fabric is supported by primary columns and cross members, configuring a pyramid shape for these membranes. Figure 2 illustrates how the stadium’s façade fabric membranes are affixed to the supporting CHS columns using steel brackets. The fabric pretensioning forces shall be transferred to the supporting steel CHS by these steel brackets which are distributed along the CHS column height. Consequently, the supporting CHS member faces a combination of both global and local stresses induced by the fabric membrane’s pre-tensioning forces. The local stresses, occurring at the joints between the steel brackets and the CHS member, stem from out-of-plane bending, shear, and tension forces induced at the conjunction of the brackets and CHS. Meanwhile, the global stresses arise from the comprehensive analysis of the overall structural framework.

**Fig. 3:**
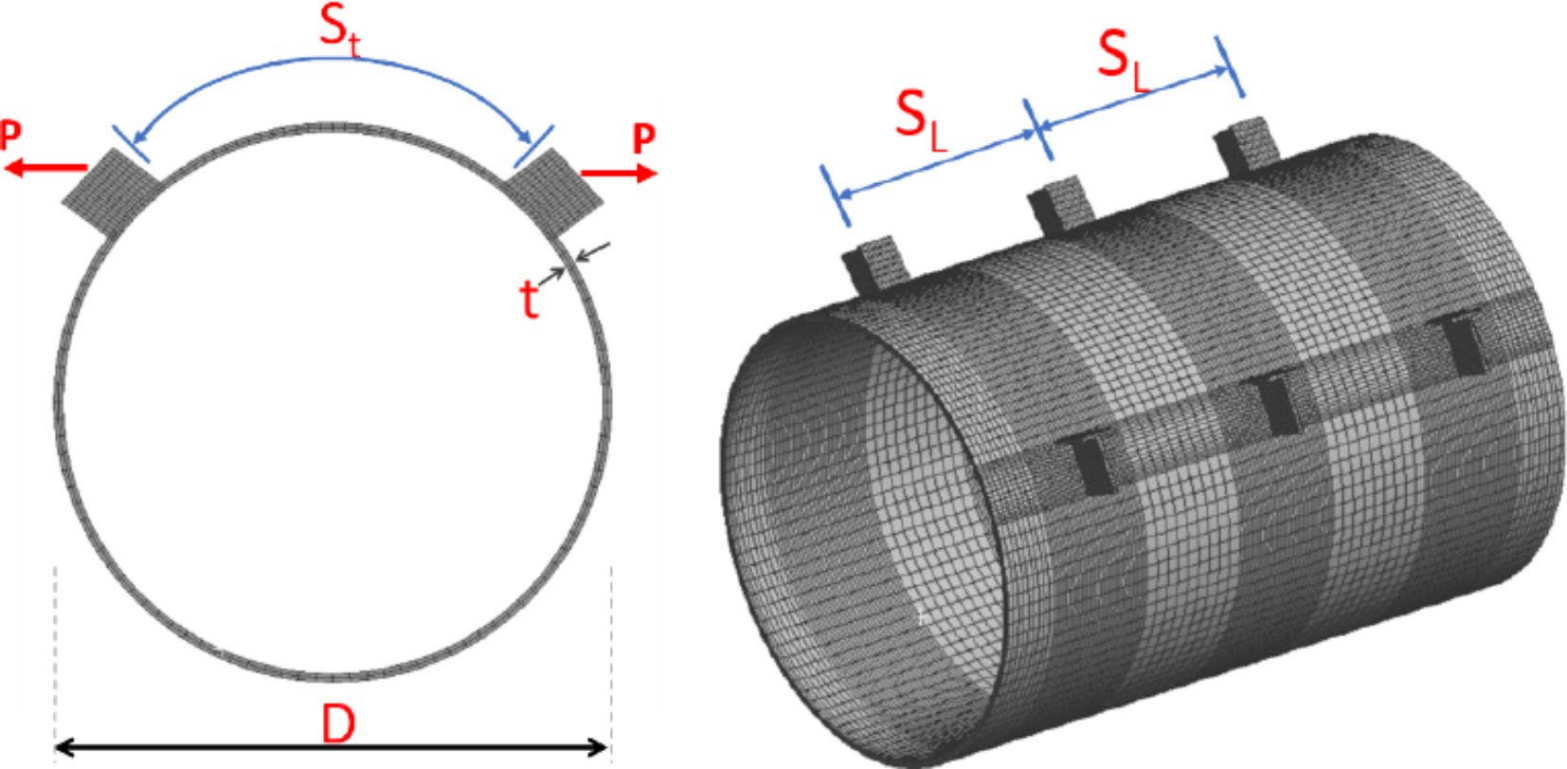
Simplified presentation for the CHS column with double brackets. (3D Modelling using Ansys v.21).

The examined joint involves CHS columns that provide support through specially designed double-bracket joints. These brackets are constructed with a unique configuration to accommodate two reeling pipes, serving the purpose of sustaining the fabric. To facilitate a more detailed analysis, the complexity of the has been simplified to double branch plate brackets, as illustrated in Fig. 3. These brackets can be utilized either with or without T-stiffeners.

The Façade main column has a large diameter (1200mm) with a large D/t ratio (120), which exceeds the AISC code limits for single branch plate configurations. The design equations’ applicability is limited to D/t ratios less than 50. However, the joint under consideration in this study has a double brackets configuration which is distributed along CHS column length. The available design codes don’t show design guidelines for such configuration.

CIDET design guide^2^ and AISC Design Guide 24^20^ provide the design limit states for single longitudinal or transverse plate to CHS joint under axial or bending loading conditions. The design capacity for such joint is always related to deformation limit rather than the ultimate strength as they normally exhibit large deformations.

Voth et al.^4,5^. performed both experimental and finite element analyses to determine the strength of single branch plates connected to CHS members under axial loading conditions. Their findings revealed that the strength of branch plate connections subjected to axial tension loads is consistently underestimated according to the design guidelines and codes. In a subsequent investigation, M. Hassan et al. ^6^ conducted experimental tests on branch plate connections to CHS under axial loading. Their results highlighted a significant reliance of connection capacity on the geometry of the connection. Addressing the compression forces aspect, Zapata et al. ^7^ introduced an analytical model for predicting the behavior of single branch plate connections to CHS members. This model utilized yield line analysis based on the theorem of plastic collapse. The proposed method exhibited a slight tendency to overestimate the connection strength. Chen, M. T. et al. ^17^ studied experimentally the behavior of cold-formed steel T-joints with semi-oval hollow section. The results indicated that the current standards are conservative. Lan, X. et al. ^11,13^ examined experimentally the strength of T- and X- joints under brace axial compression for high strength steel material. They concluded that CIDECT and Eurocode mean strength equations tend to overestimate the static strengths of T-joint specimens studied. This work was further developed in subsequent research (2021), which proposed modified equations to more accurately determine the strength of such joints.

The actual bracket is T-shaped, which improves joint behavior. Various strengthening techniques are available. Zuo et al. ^16^ tested CHS T-joints with grout-filled GFRP, while Nassiraei ^8,10,12,18^ proposed the use of collar plates to enhance the capacity of CHS X-joints and T/Y joints under ordinary conditions and various fire conditions. Fayed et al. ^19^ introduced the technique of using through bolts to enhance the strength of CHS K-joints.

According to the previous literature, the behavior and strength of double branch plates have not been studied yet. The case study for the stadium column supporting membrane is also more complicated as the configuration of double brackets distributed along the column height makes it very difficult to predict the joint strength using the available design codes. In this paper, experimental and numerical investigations have been carried out to study the behavior of such cases. The numerical analysis is conducted on identical experimental specimens and subsequently validated. The numerical results are normalized based on the same material properties to ensure a clear comparison of the results.

This research serves as a vital contribution to our understanding of the behavior of textile fabric membrane structures and the integrity of the joints within them.

## Experimental program

The experimental program comprised of eight test specimens. These specimens are designed to represent different configurations for CHS with double tensile forces.

A special test setup was designed and custom-fabricated specifically to address this issue. This specialized test setup was fabricated with the explicit purpose of testing the specimens with double tensile forces, where the loading line is located away from CHS centroid.

### Test specimens and materials

**Fig. 4:**
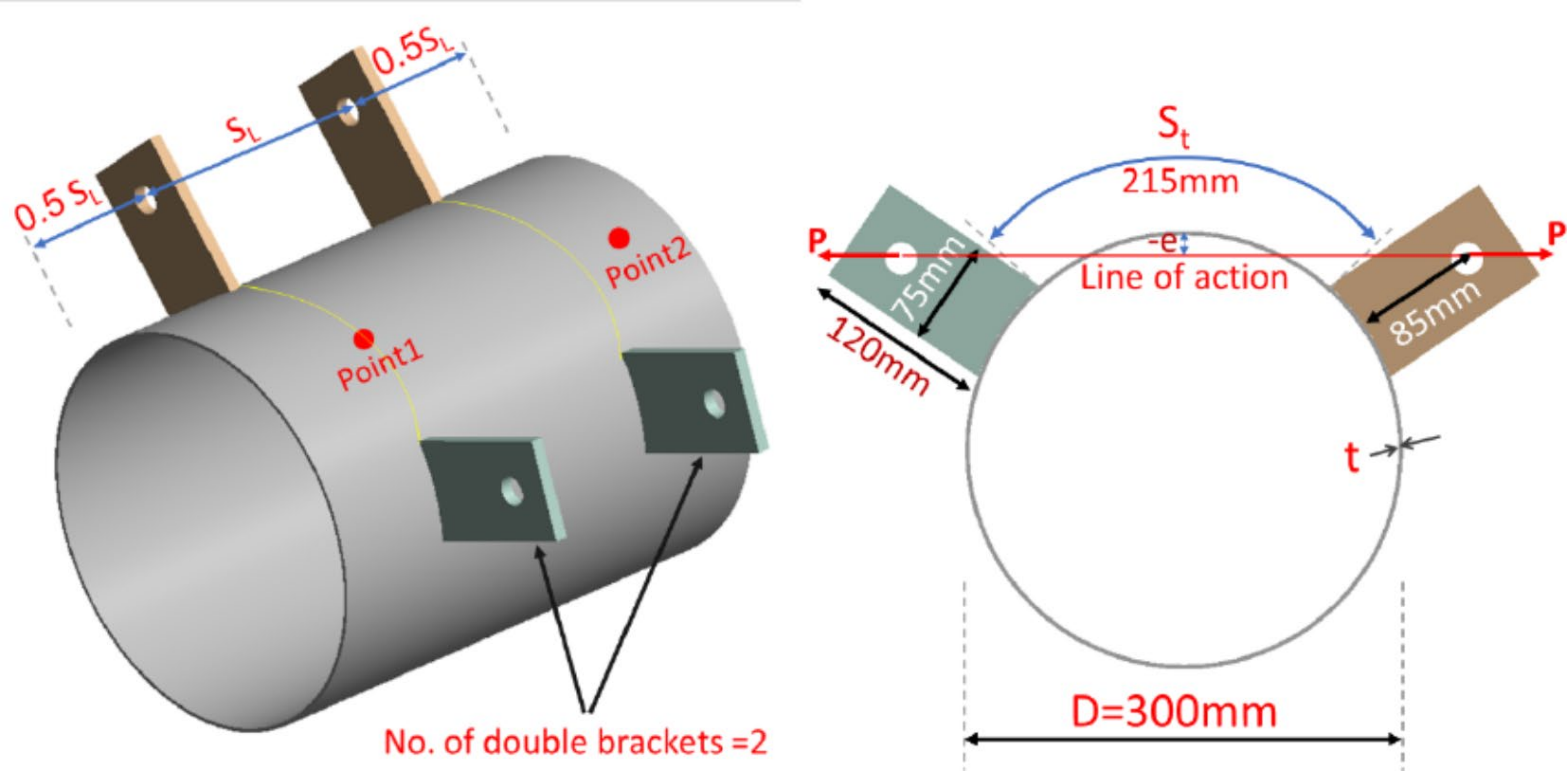
Typical definitions and dimensions of specimens. (3D Modelling using Ansys v.21).

Each specimen featured a distinct configuration, as detailed in Table 1. All specimens had a constant diameter (D) of 300 mm while thickness varies from 3 to 5mm. Specimens are indicated by S-A-B-C where “A” represents D/t, “B” for the specimen length, and “C” for no of double brackets and their special condition (T-stiffener bracket or positive eccentricity). The longitudinal spacing (S_L_) is the distance between brackets parallel to the longitudinal axis of the CHS and is equal to double the spacing between the bracket and the free end of the specimen. The transverse spacing (S_t_) is the distance along the arch between the tips of brackets within the CHS cross-section at their specific locations. All specimens share the same brackets dimensions and transverse spacing, as illustrated in Fig. 4 except the last specimen (S-100–300-1/e) where longer brackets with slightly less transverse spacing are presented. The brackets are welded using full penetration welding in order to prevent weld failure. Specimen (S-100–300-1T) adopts a T-stiffener bracket shape. The T-stiffener is present in the original column joint, necessitated by the complications of fabric fixation. The fabric holes for fixation are situated on the sides of the main branch bracket within this stiffener.

**Table 1:** Experimental program specimens.

Specimen ID	Thickness (t) mm	Length (L) mm	Bracket dimensions mm	No.of double brackets	Transverse spacing (S_t_) mm	Longitudinal spacing (S_L_) mm	Eccentricity mm
S-100–300-3	3.01	301	74.5 × 120	3	216	100	−18
S-100–400-2	3.01	400	73 × 122	2	216.5	200	−18
S-100–300-1	3.01	300	75 × 120	1	215	300	−18
S-100–400-1	3.01	405	75 × 120	1	215	400	−18
S-75–300-1	3.97	301	73 × 119	1	216	300	−18
S-60–300-1	4.99	300	72 × 120	1	216.5	300	−18
S-100–300-1T	3.01	303	75 × 121	1 + T49 × 50x8	215	300	−18
S-100–300-1/e	3.01	300	74 × 149	1	181	300	+ 21

Specimen with three pairs of brackets had initial geometric inward imperfections of 4 mm before loading due to welding of three brackets with close spacing.

The specimens exhibiting a forces line of action outside the circular cross-section of the CHS are termed as having positive eccentricity. Eccentricity (e), in this context, refers to the distance from the CHS crown point to the forces’ line of action. Except for Specimen S-100–300-1/e, all specimens feature the same negative eccentricity of 18 mm. Specimen S-100–300-1/e, however, possesses a positive eccentricity of 21 mm.

**Fig. 5:**
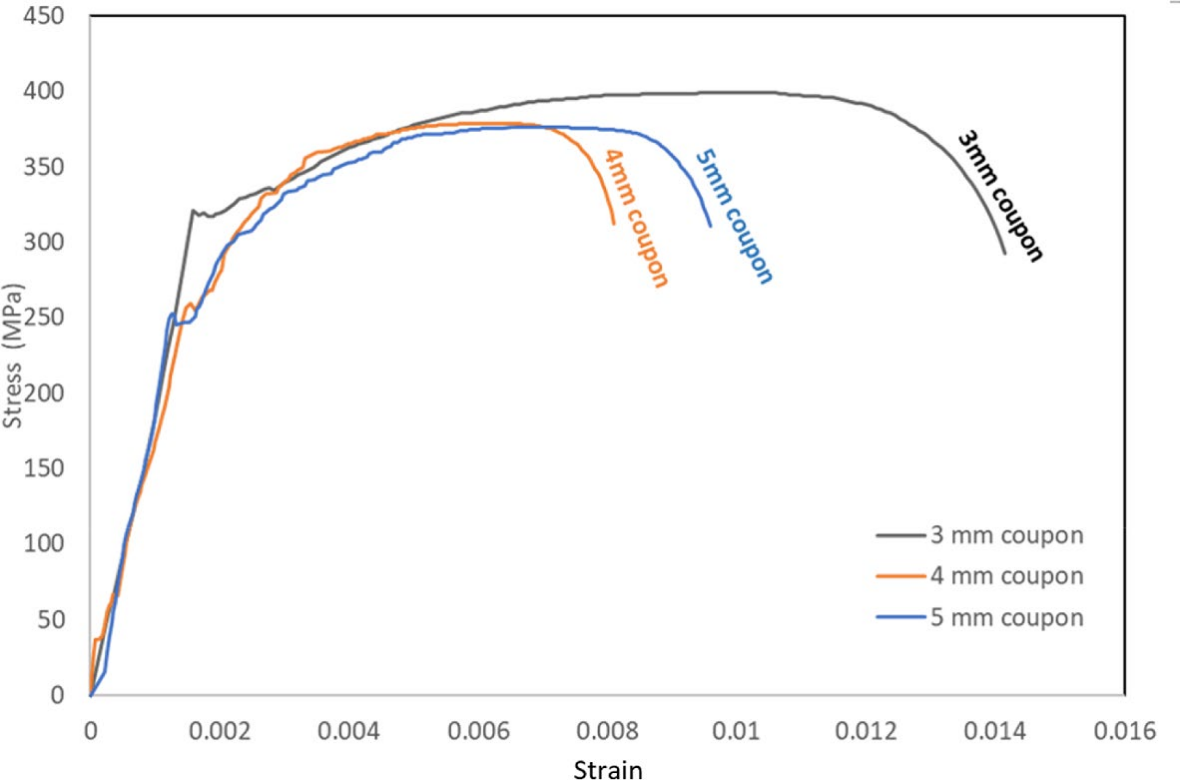
Specimens’ coupons material properties.

The material characteristics were obtained from tensile coupon tests. To assess the material properties, four coupons were tested. The 5 mm thickness material shows a higher elongation ratio and lower grade material than other tested thicknesses. The average material characteristics for the studied specimens are shown in Table 2. The stress–strain curves for the tested coupons are illustrated in Fig. 5.

**Table 2:** Material characteristics.

Thickness	3mm	4mm	5mm
Young’s Modulus E (GPa)	200	200	200
Yield Strength Fy (MPa)	320	255	245
Ultimate Strength Fu (MPa)	400	380	375

### Test setup configuration

**Fig. 6:**
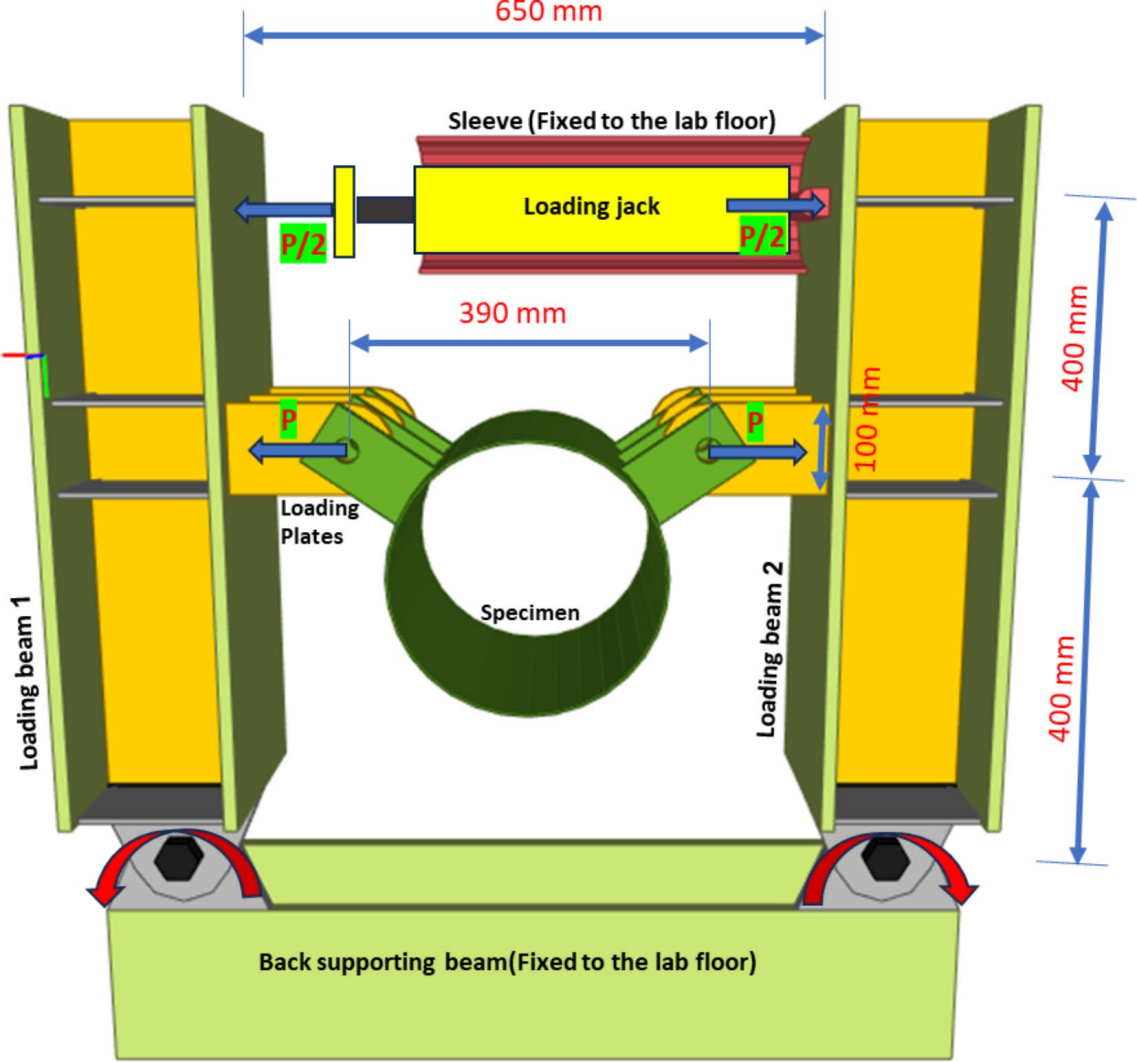
Schematic layout for test setup and loading. (Created by Advance steel 2024).

The test setup is conceptually similar to a pair of scissors, wherein the loading jack was positioned at the edge of the arrangement and aligned in position by a half-cylindrical sleeve securely affixed to a laboratory concrete raft through steel anchors. Figure 6 provides a schematic layout for the setup. The loading jack exerted forces onto two opposite loading beams, which are supported by a back-supporting steel beam at their far ends by rotatable hinges (single bolts for each beam), allowing free movement at the jack position. It should be noted that the back supporting beam is anchored to the laboratory floor using steel anchor rods.

A load cell, featuring a spherical end, was acting by loading force on the movable loading beam 1. The jack back end exerted force (via reaction force) on the other loading beam 2, facilitated by a cylindrical rod welded to the loading beam 2. The cylindrical rod and load cell ends provided roller ends to ensure opposite force directions. The design of the test setup was meticulously fabricated to seamlessly integrate with existing laboratory supports, jack dimensions, and specimen configurations. The loading beams are designed for rigidity, ensuring the efficient transfer of loads without local deformations.

The specimens were supported by the movable loading beams, precisely at the midpoint between the exerted forces and the rotatable hinges located at the beam ends. To facilitate versatility in testing various specimen configurations, three loading plates, spaced at 100 mm intervals, are welded to the movable loading beams. This arrangement enables the examination of different specimen setups.

The tensile loading on the specimens is calculated by doubling the forces recorded by the load cell.

**Fig. 7:**
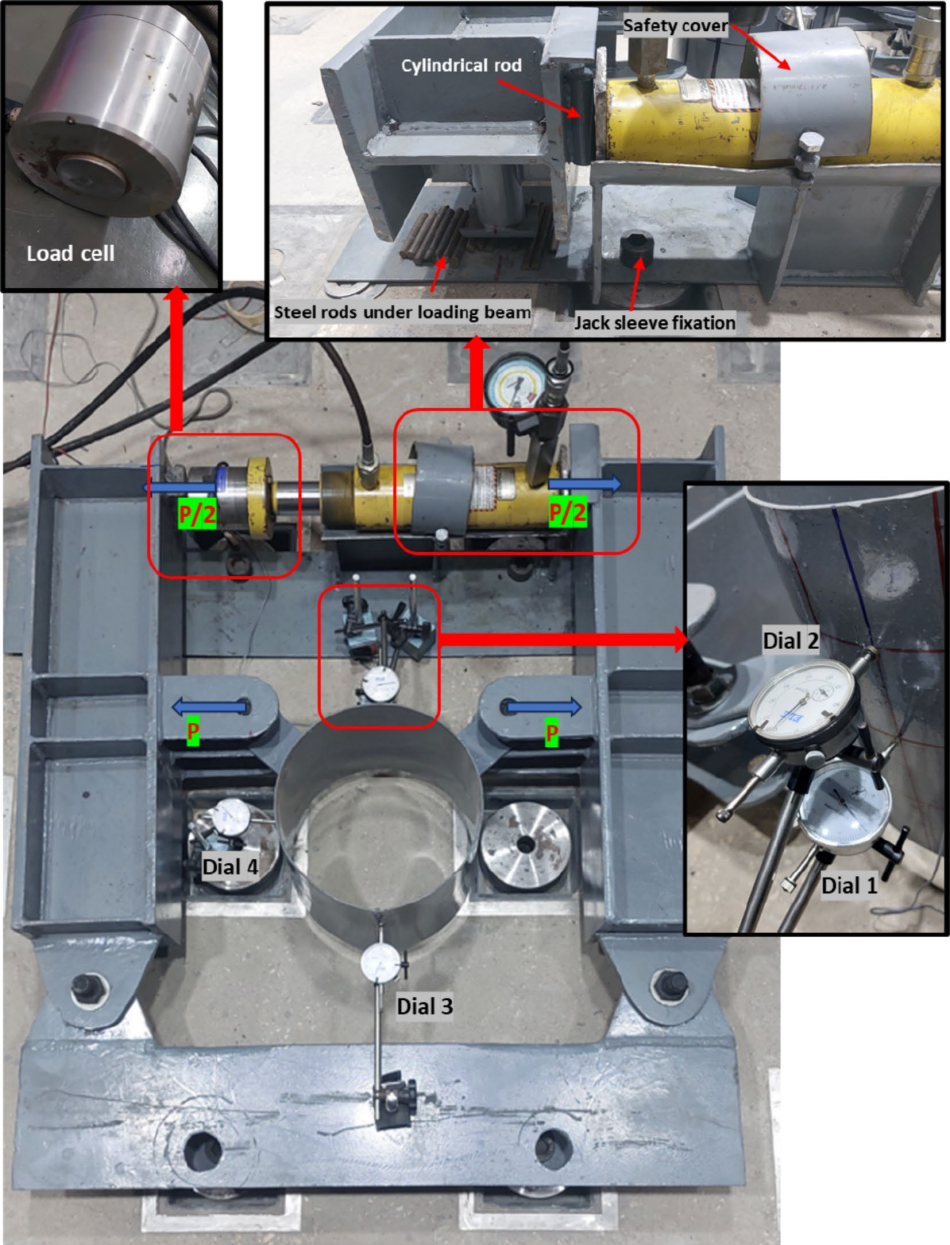
Test setup and instrumentation.

A comprehensive illustration of the entire test setup, along with its configuration and full details, is presented in Fig. 7.

For single branch plate connections, the specimen’s length should be greater than eight times the diameter to ensure that the boundary condition restraint does not affect the connection behavior. In experimental work (Voth et al. ^5^), smaller specimens are used and verified, and the connection behavior is extrapolated using numerical models with larger specimens. However, in this setup, the specimens were deliberately chosen to possess free ends. This particular configuration emulated a cut segment of the continuous case of a CHS element supporting tensile membrane fabric. The specimen with a short length and free ends is chosen to simulate the required segment, ensuring that the end conditions do not influence the results. The middle distance between the bracket plates was twice the distance between the specimen’s free end and the plate. The decision to leave the ends free with these short specimens has been validated as the most accurate representation of actual conditions and provided accurate results representing the continuously loaded brackets, as confirmed later on in the finite element analysis. This design aimed to simulate the continuity of the CHS member with multiple brackets.

### Test instrumentations

**Fig. 8:**
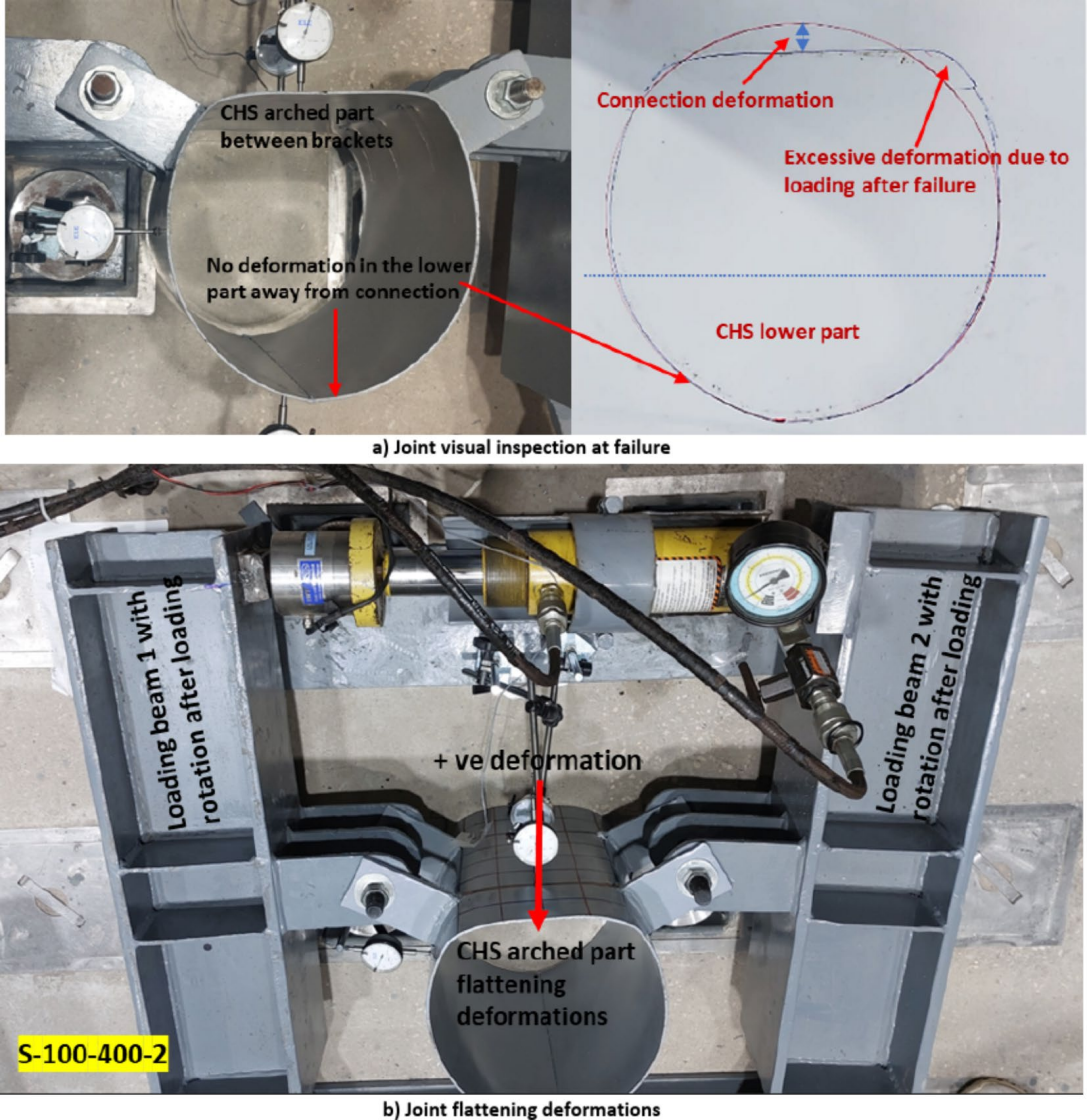
Typical deformations at failure.

Test setup instrumentations included a load cell and four dial gauges, as shown in Fig. 7. These dial gauges were employed to measure deformations, offering a range of 30mm with an accuracy of 0. 01mm. Among the dial gauges, the first one is located at the midpoint between the two brackets (point 1), while the second gauge (point 2) is positioned at the midpoint height between point 1 gauge location and the free end of the specimen. The third dial gauge is located at the rear of the CHS specimen, where no local deformation occurs in the lower part of the CHS away from the joint. However, upon loading, the entire specimen moves toward the jack position due to the setup configuration. Therefore, the combined readings of dial 1 and dial 3 represent the total deformations occurring between the two brackets. A similar approach is applied to gauge point 2 deformations. A strain gauge has been affixed at the tip of the bracket to assess stress concentration at this specific location. Additionally, the specimen is visually monitored before and after loading to confirm that all deformations are concentrated within the arched region between the two brackets as shown in Fig. 8a.

### Experimental results

The forces line of action affected the behavior of studied specimens. The behavior of all examined specimens, except for the specimen with a positive eccentricity of 21mm (S-100–300-1/e), displayed a consistent trend. The arch between the two brackets tended to flatten, with no observed deformations in the lower part of the CHS below the brackets. Consequently, all forces were primarily transferred within the arch between the brackets.

**Fig. 9:**
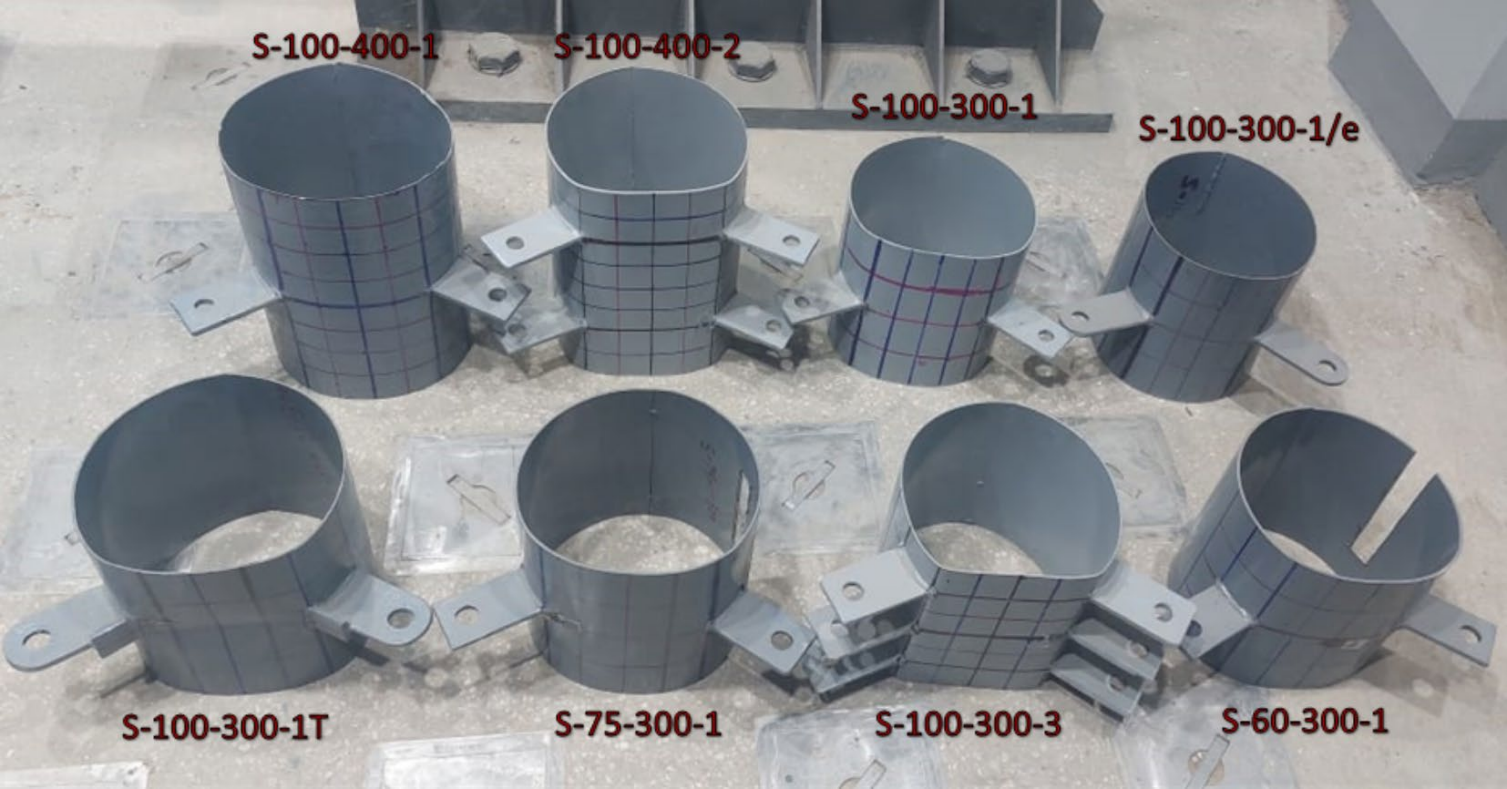
3D view for all tested specimens after deformations.

The primary deformation under consideration involves the flattening of the arched segment between the double brackets, as shown in Fig. 8b. The two key focal points associated with this deformation where the main point is the one located at the same line between the brackets, as shown in Fig. 4. A limit deformation of 3%D suggested by Lu et al. ^1^ is used in this study as a limiting criterion for these joints’ configurations. The International Institute of Welding (IIW) Subcommission XV-E^3^ adopted this limit of 3% b_0_(or 3% d_0_) as the ultimate deformation limit. Kozich et al. ^14^ proposed the strain limit of 5% ultimate strain and they found that the results for the load corresponding to a 5% maximum principal strain in a strip with a length equal to 0.5t_0_ matched the joint capacity obtained by adopting the 3%b_0_ deformation limit. The deformations between brackets exceed the 3%D deformation limit before the occurrence of fracture at the bracket tip due to excessive CHS plastification. Figure 9 illustrates the final deformed shape observed across all studied specimens.

Visual inspections have been conducted before and after loading, with documented drawings. This simple technique proved that no deformations occurred in the lower part of the CHS. Notably, during the testing of the pilot specimen (S-100–300-1), it was observed that the entire specimen moved toward the line of action while dial gauge 3 data was yet to be available. To overcome this, the results from dial gauge 2 for this specimen were compared to the total measured edge deformations for the pre-loaded and post-loaded footprint of the specimen. This comparison confirmed the necessity to add another dial gauge for the subsequent specimens.

Table 3 summarizes the results for studied specimens in terms of the brackets load obtained at 3%D deformation limit (P_3%_), the single bracket load obtained at 3%D deformation limit (P1_3%_), The brackets ultimate load obtained at failure (P_f_ ), The single bracket load obtained at failure (P1f.) and the ultimate joint deformations obtained at failure load.

**Table 3:** Summary of experimental results.

Specimen ID	P_3%_ (kN)	P1_3%_ (kN)	P_f_ (kN)	P1f. (kN)	Deformation d1 at P_f_ (mm)	Failure mode
S-100–300-3	27.9	9.3	81.2	27.07	16.00	3%D
S-100–400-2	30.6	15.3	56.0	28.0	15.44	3%D
S-100–300-1	19.72*	19.72	28.4	28.4	16.17	3%D
S-100–400-1	24.4	24.4	30.0	30.0	13.21	3%D
S-75–300-1	25.0	25.0	30.0	30.0	13.11	3%D
S-60–300-1	33.3	33.3	52.0	52.0	18.87	3%D
S-100–300-1T	25.0	25.0	N/A	N/A	16.00	3%D
S-100–300-1/e	N/A	N/A	24.8	24.8	N/A	Punching shear

*This value was obtained from finite element modeling as no data were available in the test results for this specimen.

An increase in the CHS thickness demonstrates a corresponding increase in joint capacity, particularly with regard to the 3%D criterion. It is noteworthy that specimens with greater thicknesses were fabricated from lower-grade materials. Remarkably, the fracture load values for the specimens with 3 mm thickness but varying brackets longitudinal spacing and numbers remain closely aligned. The average value of fracture load for a single bracket is 28.37 kN. The average difference for single brackets (P1f.) with the same thickness is only 5.7%. However, the strength corresponding to 3%D deformations (P1_3%_) had much higher average differences for these specimens (46.4%). This fracture load is closely aligned as the joint at failure where flattening deformations are dominant acts as a tensile direct test regardless of the load-deformation behavior that led to this failure.

#### Load-deformation behavior.

**Fig. 10:**
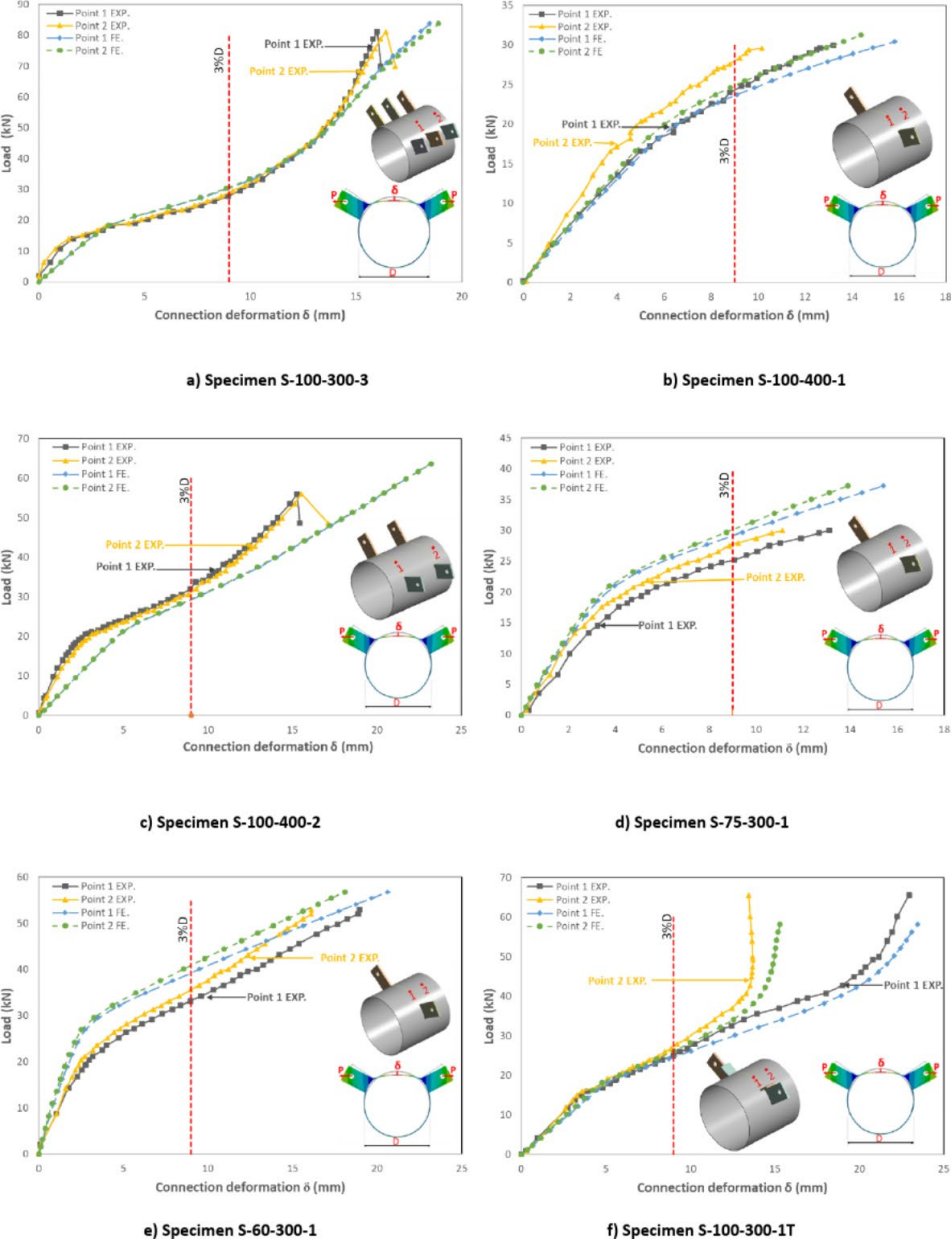
Verification of FE results. (3D Modelling using Ansys v.21).

The load versus deformation curves at the focal deformation points (points 1 and 2) are introduced in Fig. 10. For all studied specimens (except Specimen S-100–300-1/e), the load–displacement behavior could be divided into three parts. In the first part, the load increased gradually with linear behavior and slightly higher joint stiffness. A second part of nonlinear behavior with lower stiffness was then observed followed by the third part, which is mainly linear with lower stiffness. Accordingly, the load-deformation behavior for all studied specimens till the 3%D limit could be described as a bilinear behavior. The joint deformation at the observed points 1 and 2 had almost exact same results for specimens with small longitudinal spacing (ranging between 0.33D to 0.667D). For specimens with longer longitudinal spacing (ranging between D to 1.33D), the deformation at point 1 starts to have a slightly higher value than at point 2. This means that the stress interference is reduced by increasing longitudinal spacing. For specimen with transverse stiffener (S-100–300-1T), deformations at both studied points (1) and (2) exhibit a close correlation until flattening occurs between the brackets. As the loading increases, point 2 tends to stabilize and move in the opposite direction, while point 1 continues to move inward toward the CHS center, as shown in Fig. 10f.

The aforementioned behavior is not applicable for the specimen (S-100–300-1/e) as small deformations are observed, resulting in punching shear failure.

#### Failure modes

According to the aforementioned observations, the capacity of the studied joints was always determined according to the deformation limit of 3%D. This limit is followed by significant deformation of CHS leading to fracture failure due to excessive CHS plastification.

**Fig. 11:**
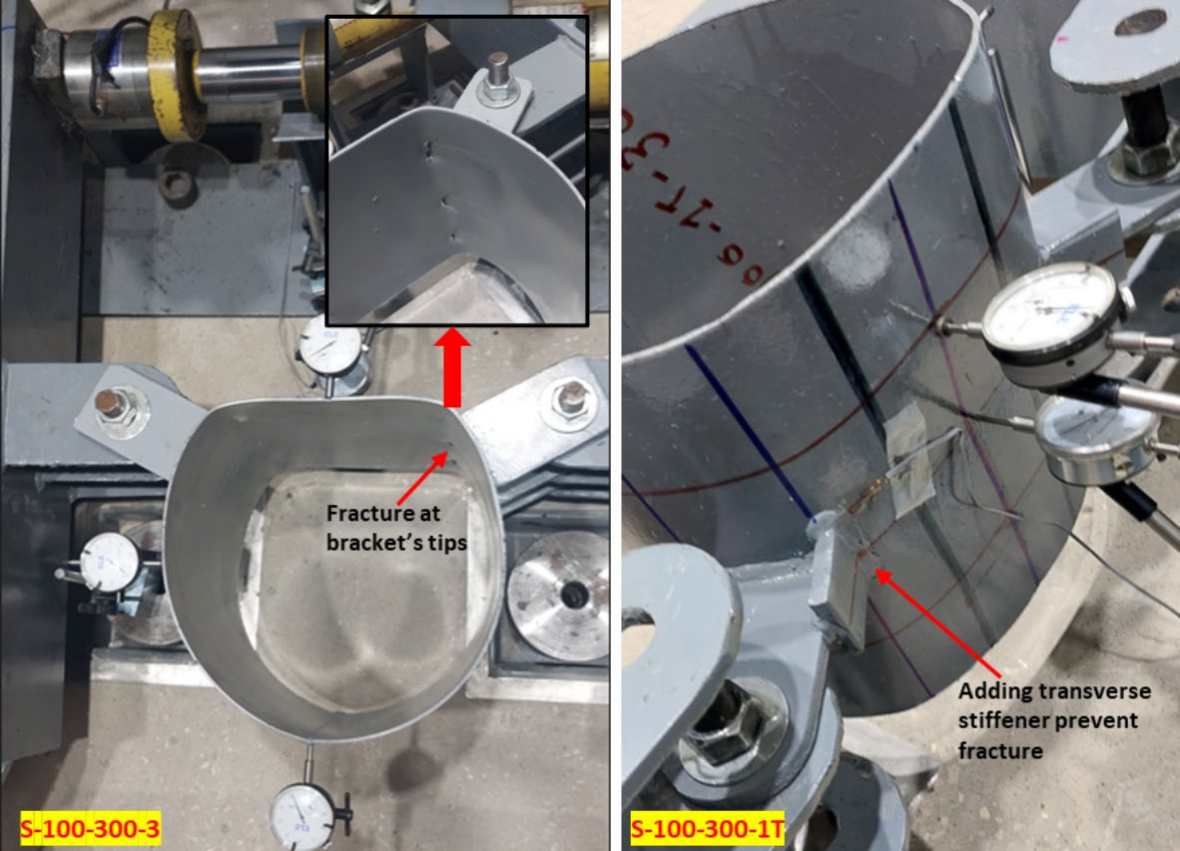
Specimens at failure.

All test specimens featuring double branch plate brackets were tested until the occurrence of fracture in the CHS member. The fracture within the CHS specimen was observed at the tip of one of the branch plate brackets, as shown in Fig. 11a. This behavior was observed in all studied specimens except (S-100–300-1/e).

Joints with additional T-stiffener exhibited excessive joint deformation without fracture or punching shear failure (until the test setup capacity 70kN), as shown in Fig. 11b.

**Fig. 12:**
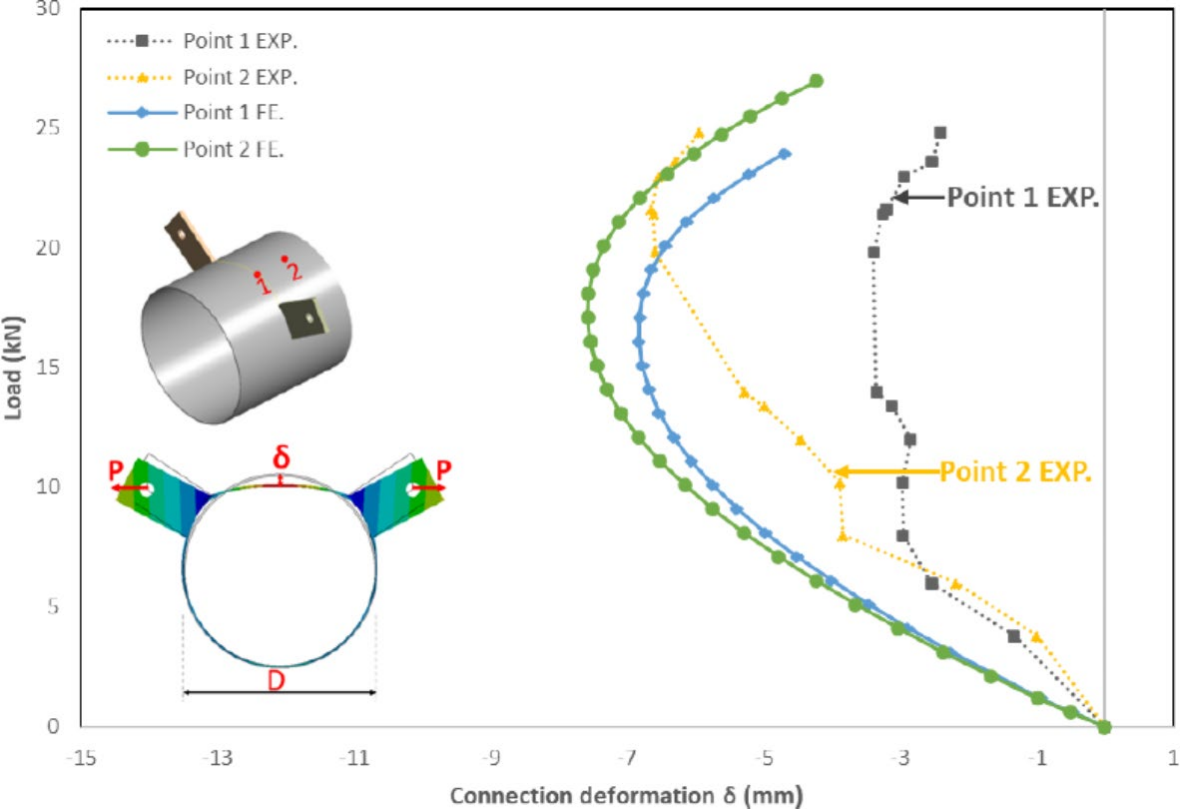
Load versus deformation for specimen S-100–300-1/e. (3D Modelling using Ansys v.21).

**Fig. 13:**
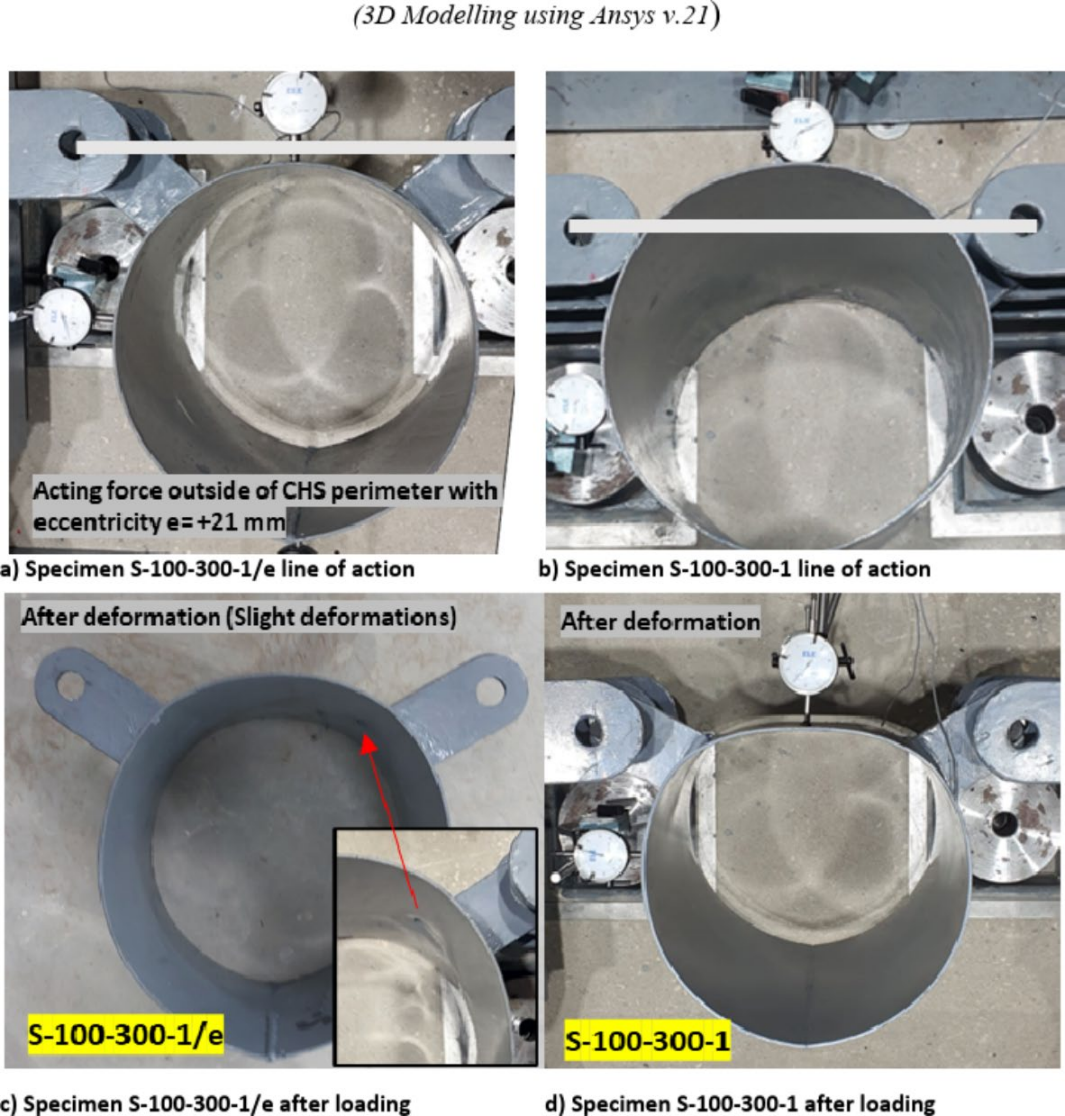
Comparison between specimens with positive and negative eccentricities.

The specimen featuring slightly longer plates (S-100–300-1/e) with the two forces outside the CHS ring (positive eccentricity of 21mm) exhibited punching shear failure with minor deformations. This specimen demonstrates a distinct behavior. In this case, the CHS experienced deformations primarily related to the rotation of the brackets, causing a distortion in the overall circular shape. The arch between the two brackets moved slightly upward toward the line of the two forces and then moved downward with excessive brackets rotation, and the final deformed shape observed with a minor overall deformation compared with the original CHS cross-section. The load-deformation behavior for this specimen was a little distorted and minor deformations were observed, as shown in Fig. 12. These small deformations are followed by a sudden punching shear. The punching shear occurs at a lower load value than the fracture loads obtained in the specimen with shorter bracket (S-100–300-1). This behavior has the benefit of a small reduction in section stiffness due to minor deformations in CHS. while at the same time sudden punching shear at brackets tips was obtained before reaching notable deformations. This behavior is shown in Fig. 13 in comparison with its counterpart specimen with negative eccentricity.

The introduction of a transverse stiffener to the original branch bracket provided a significant increase in joint strength and effectively reduced stress concentration at the bracket tip which prevented fracture in CHS until reaching the test setup capacity. The transverse stiffener has a pronounced positive impact on joint performance.

## Finite element modeling and verification

The finite element analysis was conducted utilizing the well-established ANSYS Workbench 21^21^, with modeling executed through the ANSYS SpaceClaim module. The modeling process involved a combination of solid elements, SOLID185 and SOLID186. Both elements offer features, including hyperelasticity, creep, stress stiffening, large deflection, and large strain capabilities. SOLID185 is an 8-node linear solid element, while SOLID186 is a higher-order solid quadratic element boasting 20 nodes. The latter is strategically employed in areas characterized by elevated stress or intricate geometries.

For regions with regular geometry and no connections, SOLID185 linear solid elements were utilized. Garifullin et al. ^9^ proposed that linear solid elements are suitable for regular meshes, while quadratic elements with reduced integration are more desirable for irregular and complex meshes. A multilinear stress–strain curve with a minimum of three-line segments is used to accurately represent the nonlinear behavior beyond the yield point. An isotropic hardening model is employed.

Finite element modeling was employed for the identical set of eight specimens that were experimentally studied. These specimens served as a solid basis for comparing various parameters related to the investigated joint. These parameters are summarized in Table 4.

**Table 4:** Parameters studied.

Studied parameter	Range
Diameter to thickness ratio (D/t)	60, 75, 100
Brackets Longitudinal spacing (S_L_)	0.33D, 0.67D, D, 1.33D
Implementation of transverse stiffener	Include 2T-stiffener with dimensions of 50 × 50x8
Force line of action eccentricity	e = −18 mm, e = + 21 mm

In order to obtain the proper boundary conditions, an initial study was conducted using the façade column dimensions.

### Initial study for the specimen length and boundary conditions.

The CHS column under study has a diameter (D) of 1200 mm and a thickness (t) of 10 mm, resulting in a diameter-to-thickness ratio (D/t) of 120. Both the longitudinal spacing (S_L_) and the transverse spacing (St) are set at 800 mm (0.667d). The bracket is a T-shaped section with dimensions of a width of 150 mm, and height of 125 mm, with a bracket thickness of 10 mm and stiffener of dimensions width 50mm with same bracket thickness and height.

Due to the fact that these double brackets are repeatedly welded along the CHS column height with a spacings less than the column diameter (D), an initial study was conducted to ascertain the suitable CHS column length for practical representation of specimen behavior. Five different specimens were considered, varying in column length, number of brackets in the longitudinal direction, and end conditions to determine the most appropriate size for simulating the real scenario.

The modeling approach employed for the double bracket joint followed the methodology detailed in the preceding section. The material selected for this analysis is S355, characterized by a yield strength of 360 MPa and an ultimate strength of 576 MPa which represent the material used for façade columns inspiring our study. The applied force is directed in the X-direction, aligning with the actual orientation of the pretensioning force transferred from the fabric membrane. The bracket is represented as a simplified T-shape bracket. The primary deformation under consideration is the downward movement of the crown point of the CHS column.

Significant deformations were observed as the specimen tended to flatten between the two brackets.

**Fig. 14:**
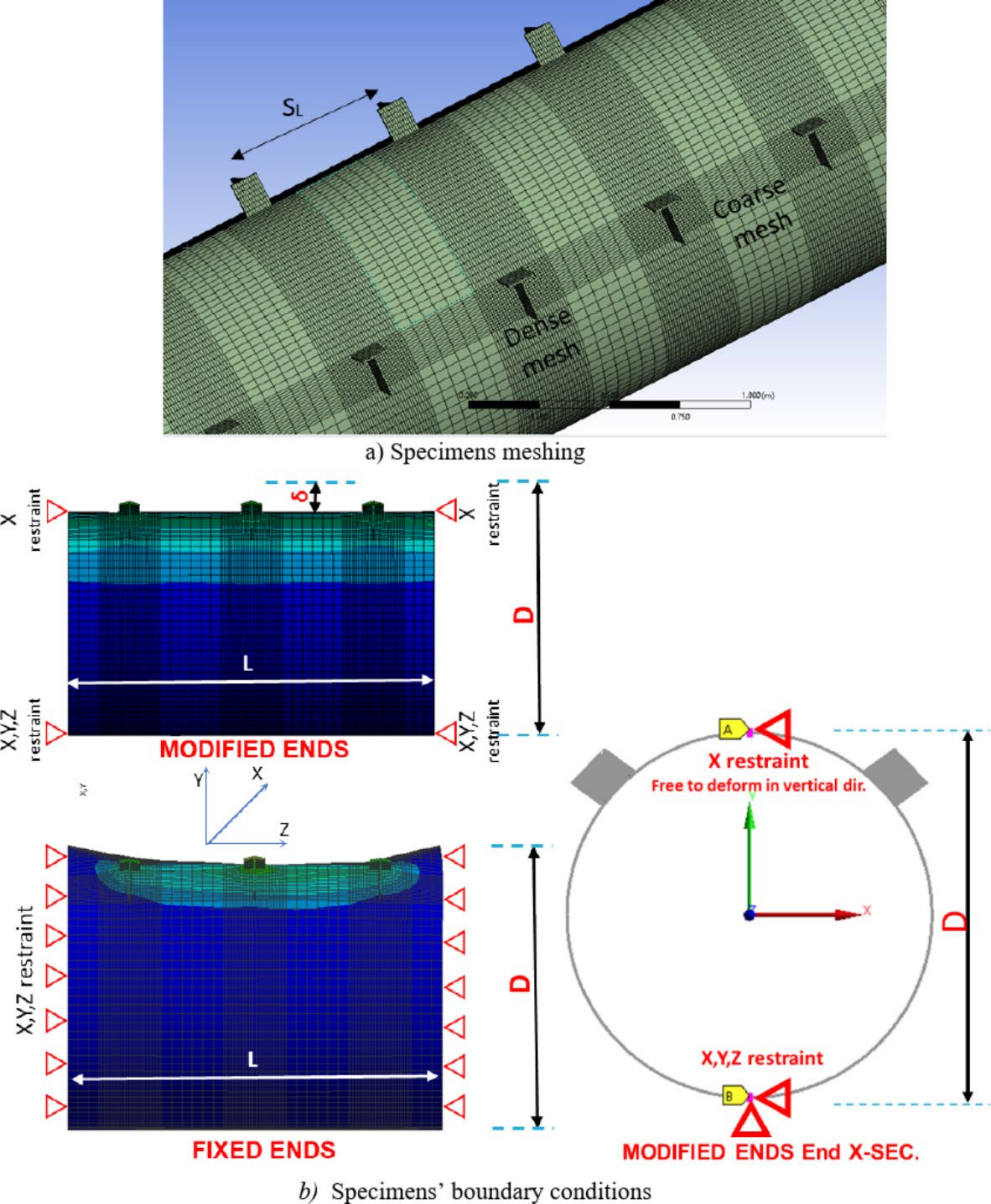
Meshing and boundary conditions. (3D Modelling using Ansys v.21).

To optimize the meshing, the mesh size ranged from 5 to 10 mm. A dense mesh is implemented at the junction between the brackets and the chord, ensuring accurate representation of critical areas. Conversely, coarser meshing elements are applied to the straight segments of the chord, promoting computational efficiency, as illustrated in Fig. 14a. Two elements are used through the thickness of the CHS.

**Fig. 15:**
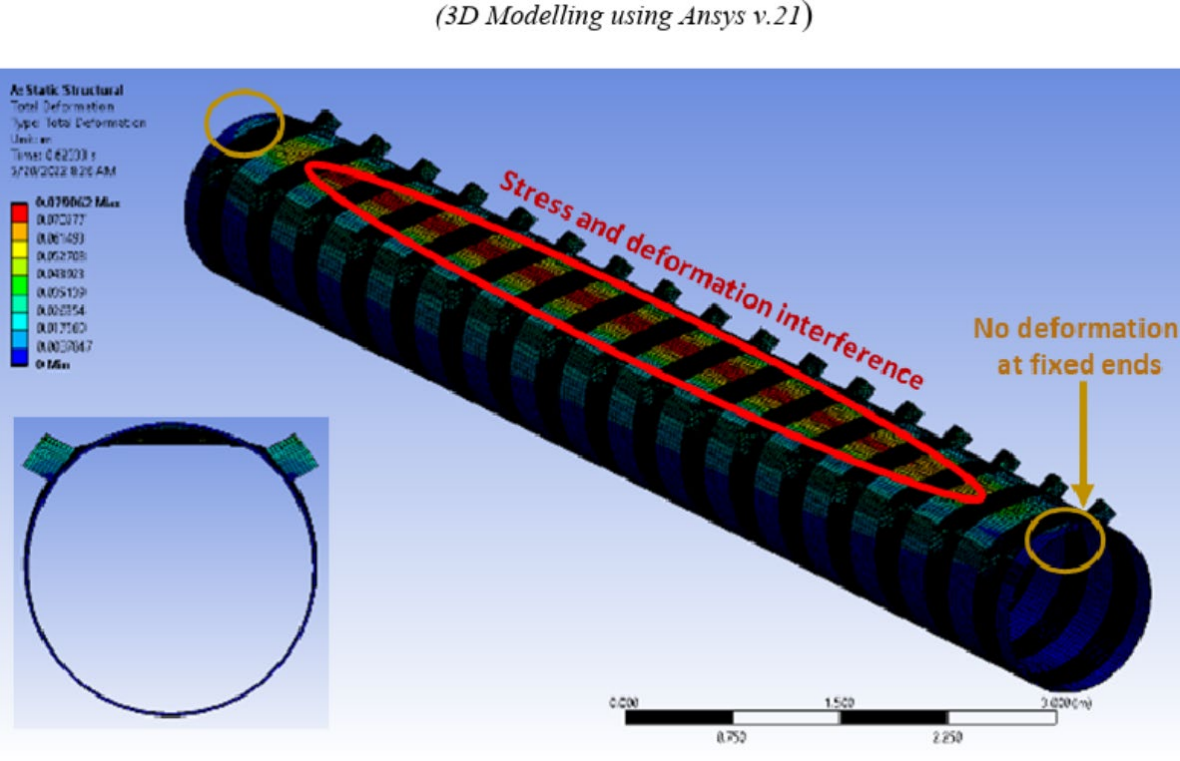
Deformed shape for specimen A17-F with fixed boundary conditions (3D Modelling using Ansys v.21).

Among these, a long specimen (A17-F) adopted fixed boundary conditions, restraining the CHS column against vertical and horizontal displacements at the end faces. The specimen featured 17 double brackets distributed along the CHS length (Length > 8d). Analysis indicated that the results for the middle double brackets (brackets no. 8, 9, & 10) were similar and did not significantly overlap with the boundary conditions, as shown in Fig. 15. Due to the computational demands and time constraints associated with analyzing such long specimens. Another four specimens (A17, A9, A3 and A1) are constructed. Specimen (A17) mirrored the configurations of specimen (A17-F) featuring the same length and number of brackets. Fewer brackets numbers were employed for the remaining three specimens, all sharing the same configuration. However, these specimens adopted modified boundary conditions that emulate the continuity of the column from both ends. At the crown points of the CHS member, lateral supports are applied at its ends, while lateral and vertical supports are employed at the invert points at the member ends, as illustrated in Fig. 14b. To ensure stability, longitudinal supports are applied at one end.

**Fig. 16:**
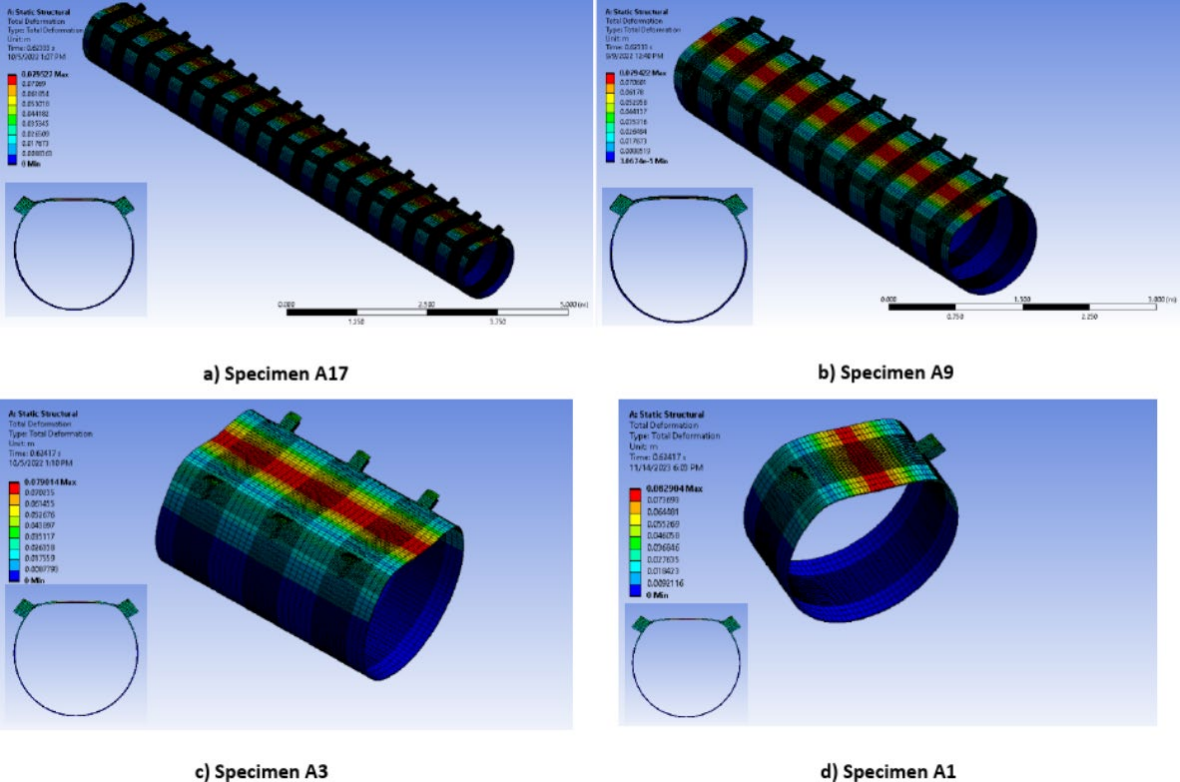
Deformed shape for specimens with modified boundary conditions. (3D Modelling using Ansys v.21).

**Fig. 17:**
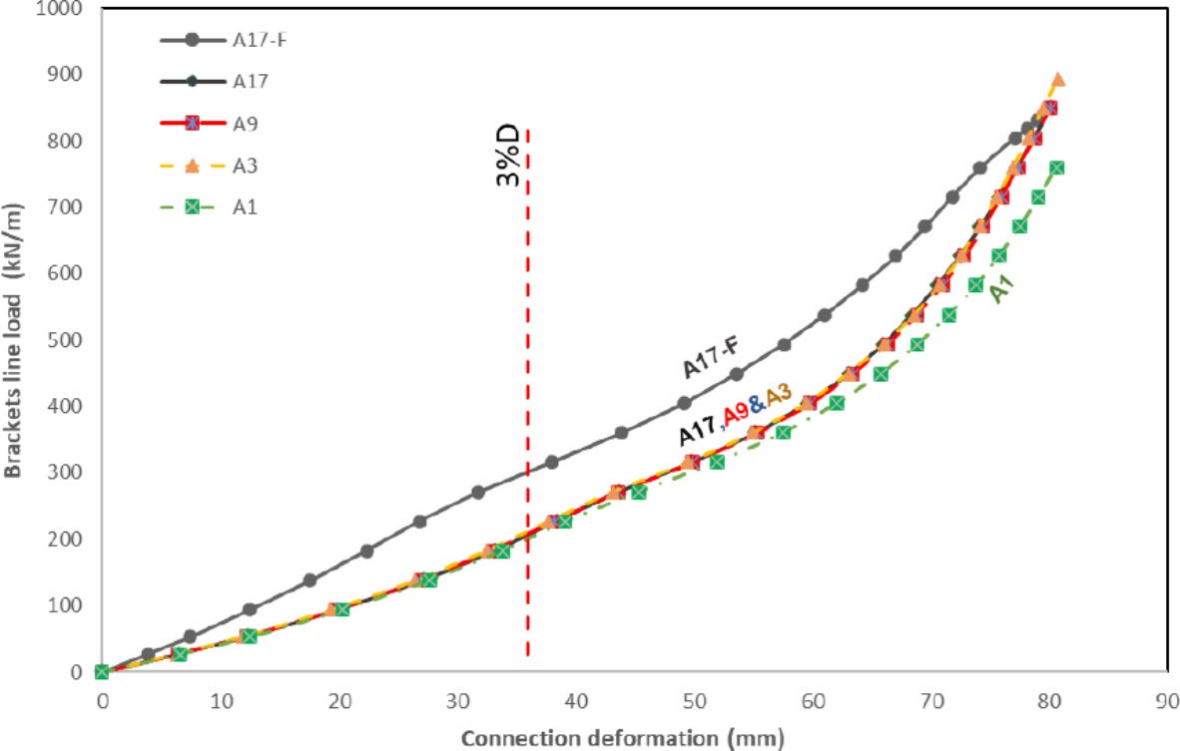
Load versus deformation for specimens with different lengths and end conditions.

Consistent behavior is observed across these four cases, as illustrated in Fig. 16. The results from Specimens A17, A9, and A3 with modified end supports are closely aligned. Additionally, Specimen A1, featuring modified ends and a single double brackets configuration, exhibited slightly smaller results compared to the other specimens , as demonstrated in Fig. 17.

The specimen A17f., under fixed boundary conditions and considering the middle double brackets, exhibited a capacity of 308 kN/m. This value surpassed the capacities observed in other specimens with modified boundary conditions, which measured 210 kN/m. This confirmed that up to a length of L > 8D, the fixed boundary condition continues to impact the results for middle brackets. This results proved that specimens with modified end conditions presented the most suitable configuration for studying the behavior of the joint. These end conditions shall be used in the verification of the experimental work.

This preliminary study provided compelling evidence that the utilization of single brackets with free end conditions, as implemented in the aforementioned experimental work, is considered a suitable approach for testing joints of this nature.

### Finite element verification for double bracket to CHS joints

Finite element models are established considering the same techniques and modified end conditions, as established in the initial study. The specimens are smaller than the actual column studied initially. Accordingly, the mesh size ranged from 1.5 mm to 5 mm, with denser meshing at the bracket locations and a coarser mesh away from the joint. Two layers of elements are used through the CHS thickness. The results of the sensitivity study for specimen S-100–300-3, evaluating the impact of different mesh sizes and layer configurations, are summarized in Table 5. The mesh sizing in longitudinal direction was always larger than sizing in transverse direction.

**Table 5:** Sensitivity analysis for mesh sizing.

Size at plain areas (mm)	Size around connection junction (mm)	Size through thickness (mm)	Elapsed time for analysis (min.)	P_3%FE_ (kN)	P_3%EXP._ (kN)	P_3%FE_ / P_3%EXP_
10 × 20	4 × 10	3	31	32.14	27.90	1.1519
7 × 15	3 × 7	3	39	32.25	27.90	1.1559
7 × 15	3 × 7	1.5	42	31.275	27.90	1.1209
5 × 10*	2 × 5*	1.5*	53	30.30	27.90	1.0860
3 × 5	2 × 5	1	107	30.31	27.90	1.0864

*The chosen mesh sizing.

In the finite element modeling of the joints, lateral and longitudinal movements were restricted in far points located in the lower part of CHS, facilitating the direct measurement of the joints deformation. This value is then compared to the joint deformation obtained in the experimental work by summing the readings of dial 1 and dial 2 to dial 3. While the supports utilized in the models, designed to represent continuity, exhibit nearly zero reactions, the program necessitates complete stability for accurate solution. Except for the end conditions, the finite element verification models are constructed using the same dimensions, materials, imperfections (S-100–300-3), and configurations, replicating the specimens used in the experimental work.

**Fig. 18:**
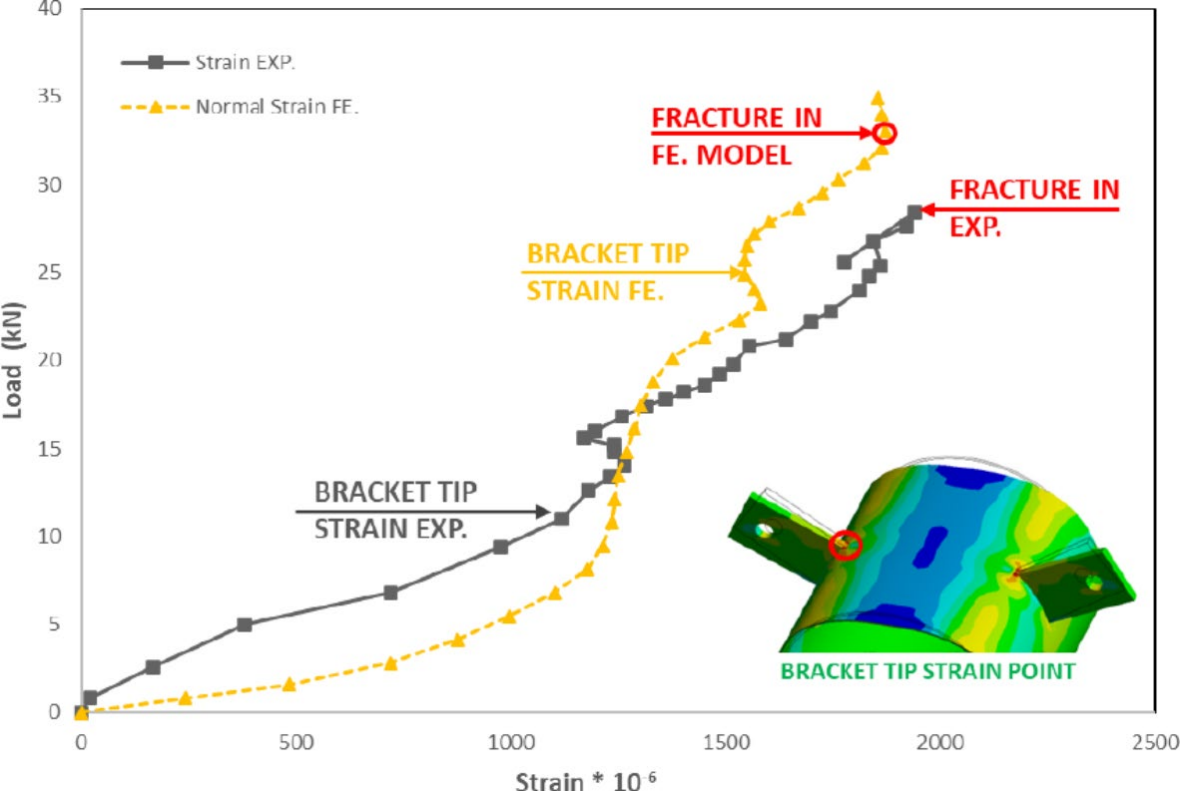
Bracket tip strain for specimen (S-100–300-1). (3D Modelling using Ansys v.21).

**Fig. 19:**
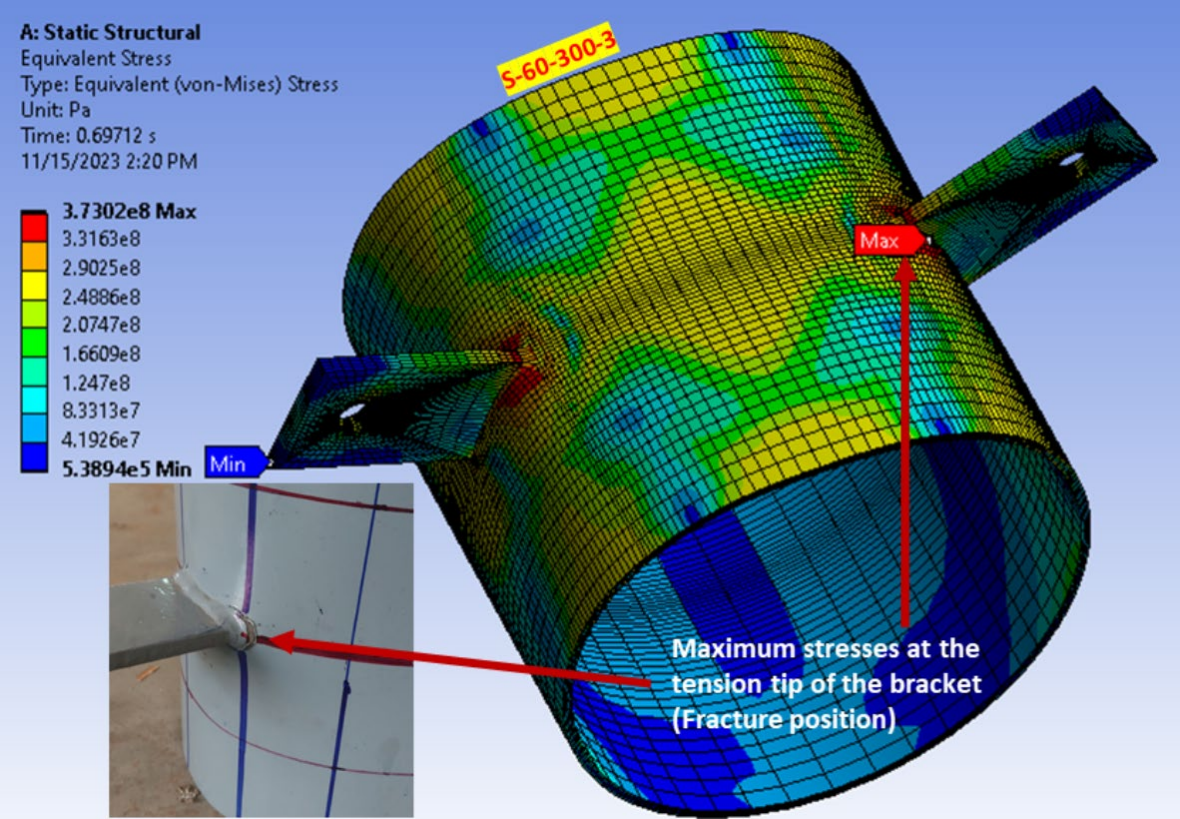
Fracture at maximum stressed area. (3D Modelling using Ansys v.21).

The FEM considered fracture point detection by identifying the highest normal strain measured at a point away from the branch plate tip by a distance equal to half the thickness of CHS, as shown in Fig. 18. The localized strain values observed are quite small, measured in micro-strain (10⁻⁶). The figure illustrates a close trend in the load strain results, with some discrepancies. These discrepancies could be resulted from simplified material models and the complex stress distribution, particularly at the bracket tip, can contribute to these discrepancies and the unusual trends observed. This location at the tension side of the bracket connection tip exhibited the maximum stresses where the fracture occurred in all specimens as shown in Fig. 19.

**Fig. 20:**
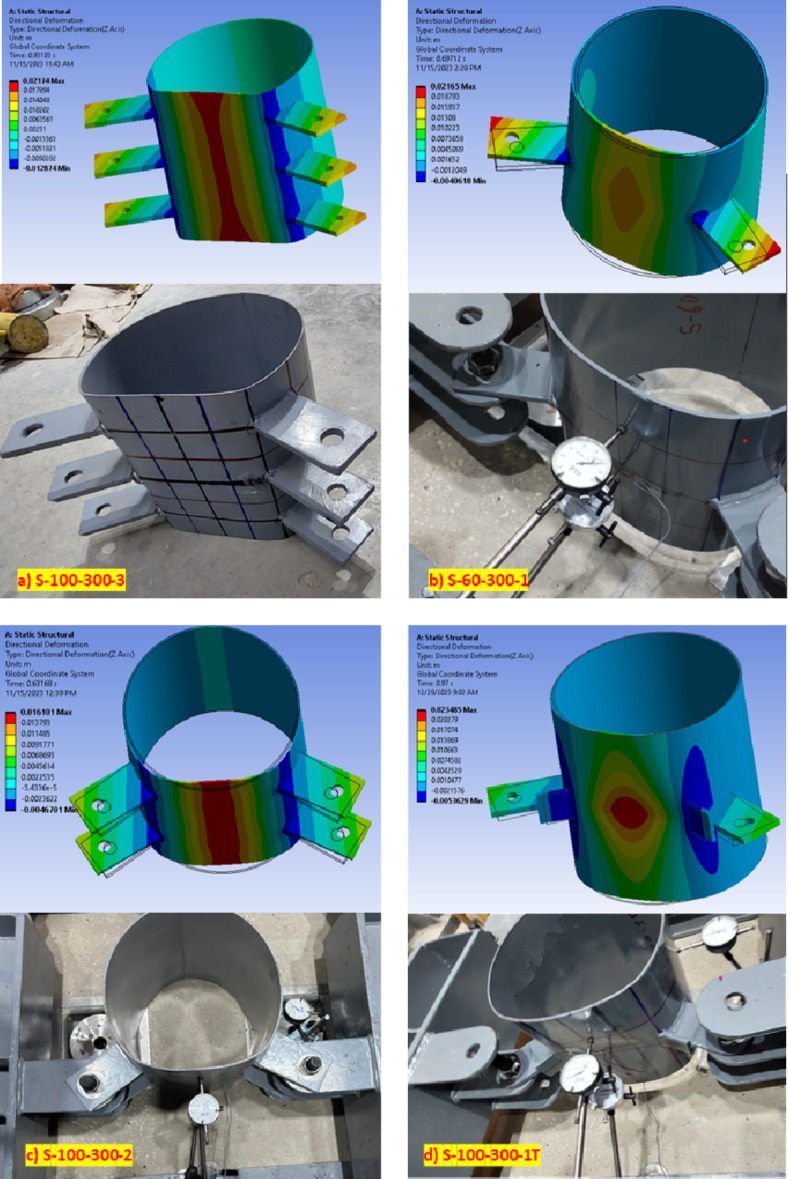
Verification of deformed shape at failure. (3D Modelling using Ansys v.21).

The finite element models provided a strong agreement, as demonstrated by the load versus deformation curves depicted in Fig. 10 when compared with experimental results. Figure 20 illustrates that the same behavior observed during experimental tests is replicated in the finite element models.

The maximum load capacity considered in the verification is that corresponding to 3%D at point (1) representing the CHS ovalization limit considering that no lateral movements observed at CHS sides. The summary verification results are shown in Table 6.

**Table 6:** Experimental load capacity versus finite element analysis results.

Specimen	P1_3%EXP._ (kN)	P1_3%FE_ (kN)	P1_3%FE_ / P1_3%EXP_
S-100–300-3	9.30	10.10	1.086
S-100–400-1	24.40	23.70	0.971
S-100–400-2	15.30	14.67	0.959
S-75–300-1	25.00	29.48	1.179
S-60–300-1	33.30	39.11	1.174
S-100–300-1T	25.00	24.90	0.996
Average Ratio			1.068 **(6.8%error)**
Standard deviation			**0.10**

A discrepancy was observed between the finite element model and the experimental results for specimen (S-100–400-2). This difference may be attributed to stress concentrations that occurred during specimen fabrication or any additional imperfections which were not observed in specimen’ measurements. However, the results were close at the 3%D ultimate limit considered.

Specimens (S-100–75-1) and (S-100–60-1) models had similar behavior as the experimental results. However, the point of inflection in the finite element models is higher than the corresponding experimental results. Furthermore, in the load deformation curve of the experimental specimens, point (2) demonstrates a slightly greater stiffness than point (1) while the same load deformation behavior is obtained in both points in the finite element results.

This disagreement in results suggest that the yielding point in the experimental specimens may be influenced by the welding of brackets, resulting in material lower yield points at the bracket to CHS junctions.

This observed behavior contributes to an increased capacity in the finite element models, particularly concerning the 3% deformation limit.

## Finite element results comparison.

The finite element verification models exhibited a commendable alignment with the experimental results with an average error not greater than 6.8%. For a comprehensive presentation of the studied parameters, all specimens are standardized with the same materials, and no imperfections are considered across any specimens.

**Fig. 21:**
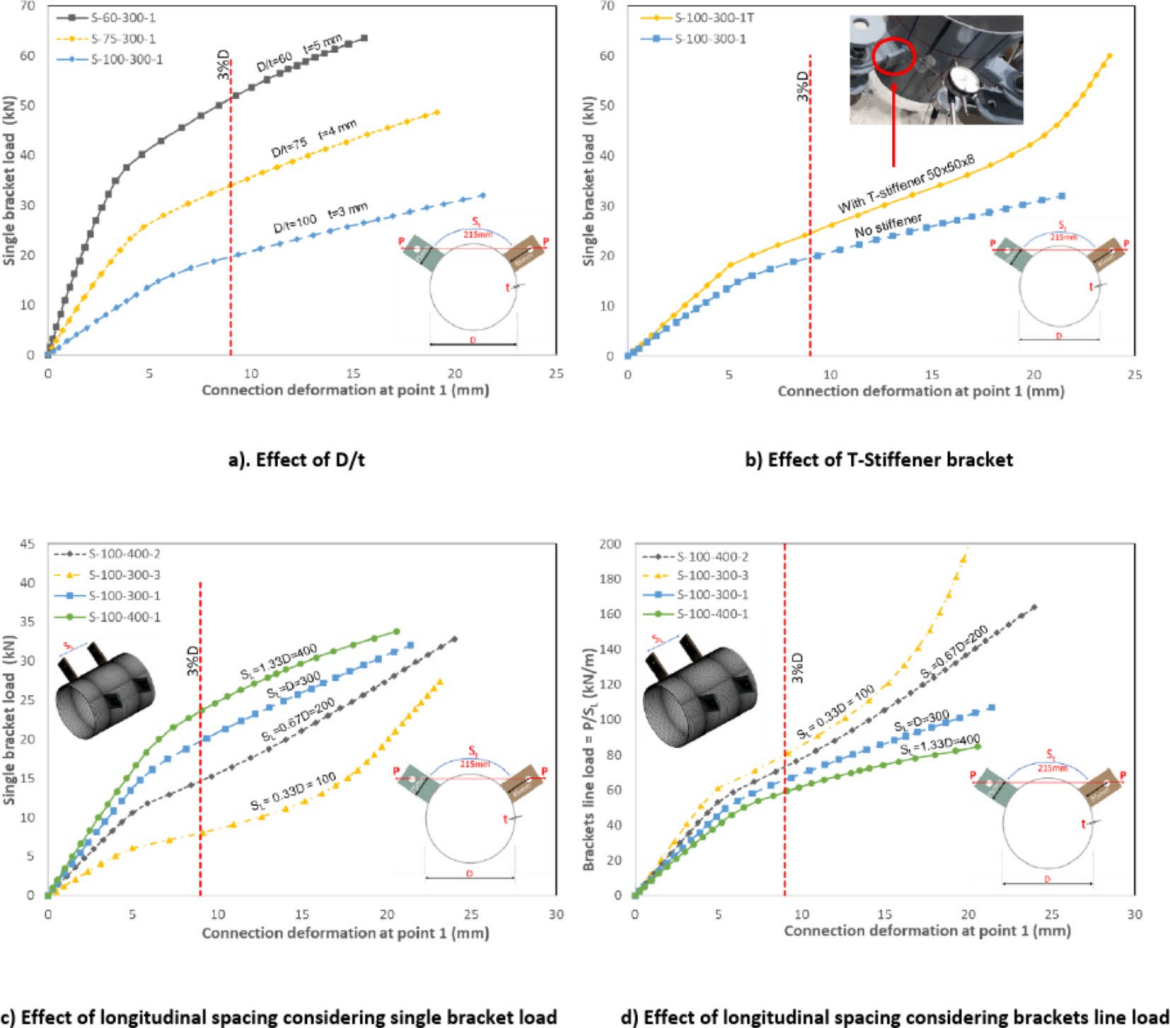
Load versus deformation for specimens considering different parameters. (3D Modelling using Ansys v.21).

The load–displacement curves at the crown point between brackets (point 1) considering different parameters are illustrated in Fig. 21.

### Effect of diameter to thickness (D/t) ratio.

The three examined specimens featuring different thicknesses are compared, with the consideration of normalizing the material properties to match those of specimens to be the same as 3mm thickness specimens’ material.

**Fig. 22:**
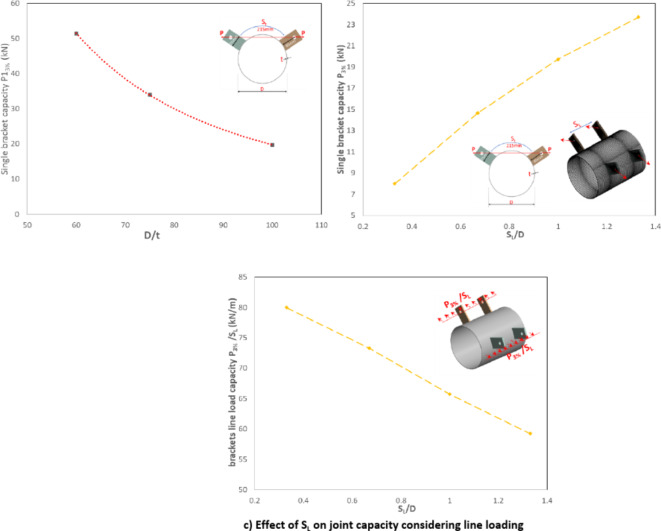
Effect of D/t and S_L_ on joint capacity (P_3%_). (3D Modelling using Ansys v.21).

The joint strength, taking into account the 3%D deformation limit, exhibited a significant dependence on the D/t ratio. Specifically, the joint strength (P1_3%_) increased by 160% for D/t = 60 compared to D/t = 100, as shown in Fig. 22a.

Bilinear behavior is observed for different D/t under examination. The big enhancement in joint strength is achieved primarily as a result of increased stiffness with lower D/t ratio, particularly in the initial linear phase of the joint’s behavior.

### Effect of adding transverse stiffener (T-stiffener effect)

The specimen featuring an additional T-stiffener added to the bracket configuration is compared to its counterpart without a stiffener.

During the experimental test, the specimen with the stiffener demonstrated remarkable better resistance, showcasing no signs of fracture or punching shear, a phenomenon observed in specimens lacking the stiffener.

Moreover, the presence of such stiffener significantly enhanced the joint strength (P1_3%_) by almost 25%.

### Effect of longitudinal spacing (S_L_).

**Fig. 23:**
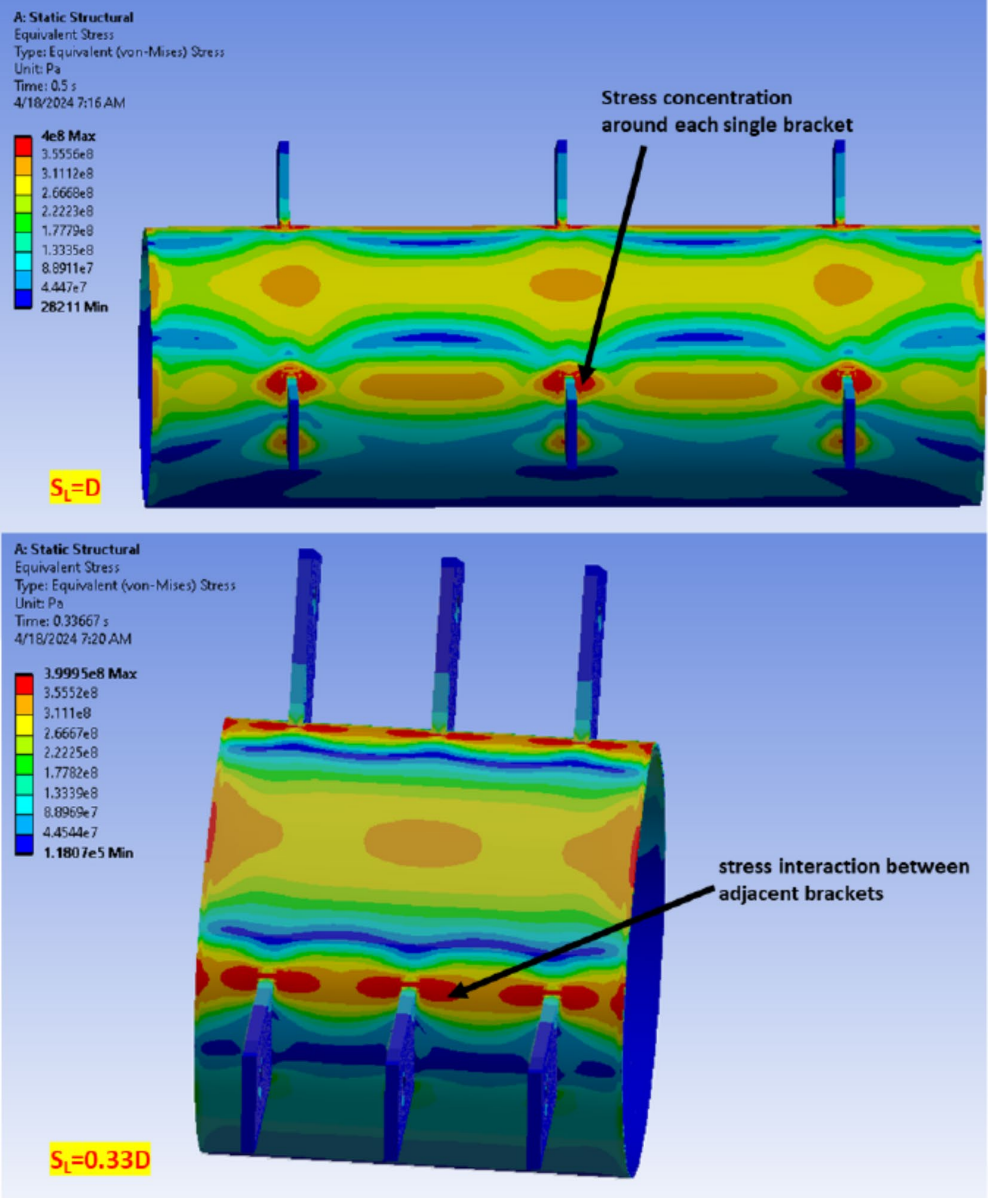
Stresses overlap between brackets. (3D Modelling using Ansys v.21).

The joint strength, as evaluated by the load on a single bracket, increased with the increase of the longitudinal spacing between brackets, as shown in Fig. 22b. However, this result may not be entirely accurate, given the nature of changing the longitudinal spacing itself. To address this, the single bracket load is normalized by dividing it by the longitudinal spacing, transforming it into a line load. This normalization allows for a more meaningful comparison of the joints brackets’ line load, revealing that the joint capacity regarding line loading is slightly decreasing as the longitudinal spacing is increased, as shown in Fig. 22c. The line loading capacity for specimen with big longitudinal spacing S_L_ = 1.33D is lower than specimen with S_L_ = 0.33D by 26%. This behavior indicates that higher stress concentration is obtained around single bracket as the longitudinal spacing increases. More adjacent brackets with smaller longitudinal spacing lead to higher stiffness as brackets act as line stiffeners for the CHS leading to better line loading performance, as shown in Fig. 23. However, higher stress concentration between brackets is observed in these cases.

### Effect of load eccentricity

The specimen featuring a slightly larger eccentricity of + 21 mm, where the double brackets force line of action fall outside the circle cross section of the CHS, exhibited a distinct behavior. While all other specimens exhibiting negative eccentricity display a different behavior. This particular specimen displays lower punching shear strength, accompanied by minimal deformations in the CHS specimen as mentioned before in the experimental results. Specimens exhibiting negative eccentricity demonstrate a force transfer within the arched area between brackets. This force transfer persists through significant deformations until the arched area is fully flattened.

The load deformation curve for finite element models for specimen with e =  + 21 mm was more consistent than the experimental behavior observed. However, results were close as minor deformation less than 3%D were obtained, as illustrated in Fig. 12.

**Fig. 24:**
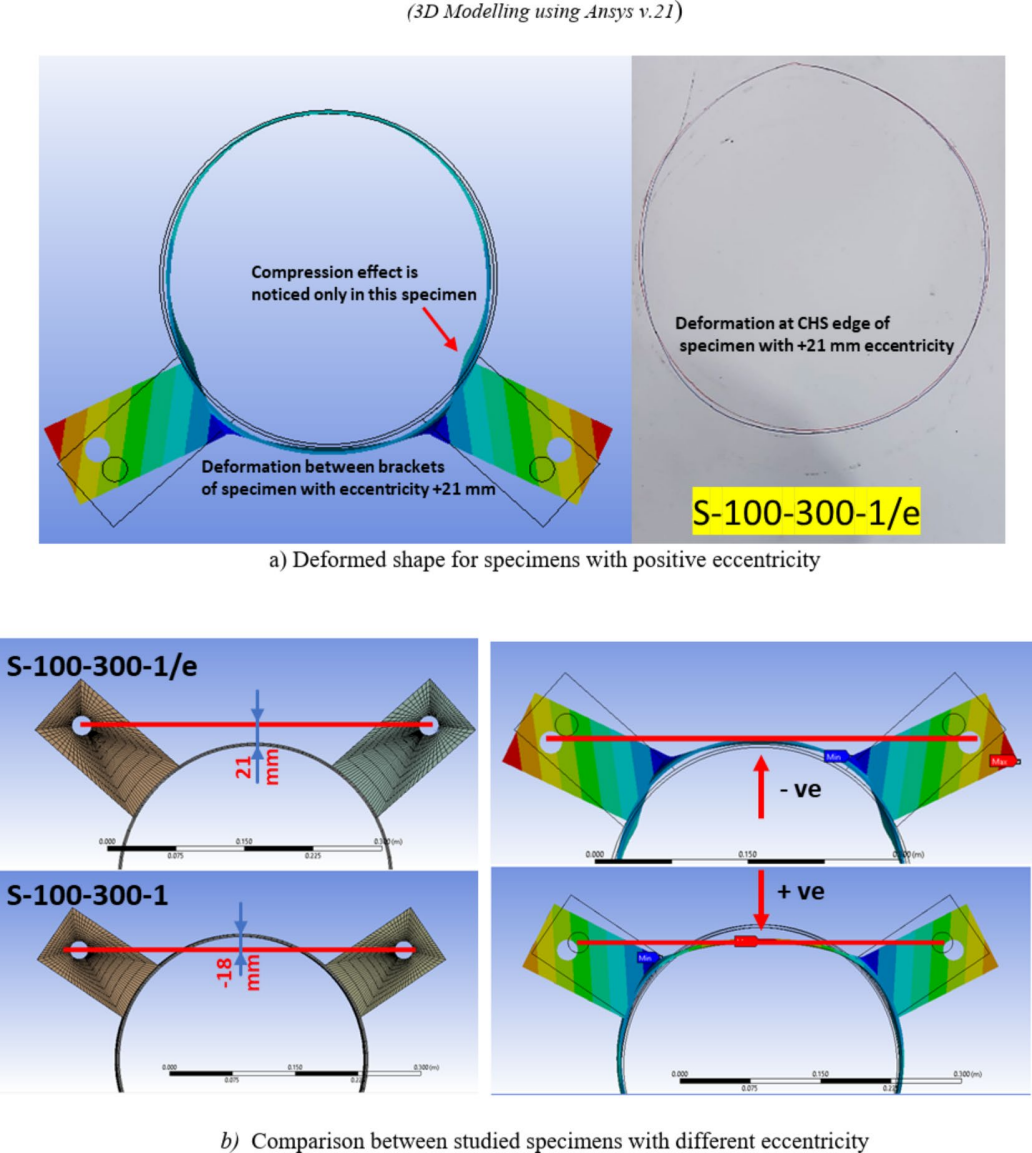
Effect of eccentricity direction on the joint behavior. (3D Modelling using Ansys v.21).

The findings for this specimen, characterized by such behavior, are further confirmed by the finite element model designed specifically for this configuration, as shown in Fig. 24, the behavior of this specimen is clearly illustrated, allowing for a direct comparison with its counterpart specimen (S-100–300-1) without this positive eccentricity, where the forces are transferred in the arched area between brackets.

## Proposed strength equation for double bracket-to-CHS column joints

The X-type joints are the closest configuration to the double bracket joints. For X-type joints, the two forces are aligned along the same line with a 180-degree angle. However, the two brackets of the joints studied in this research introduce an internal angle less than 180.

**Fig. 25:**
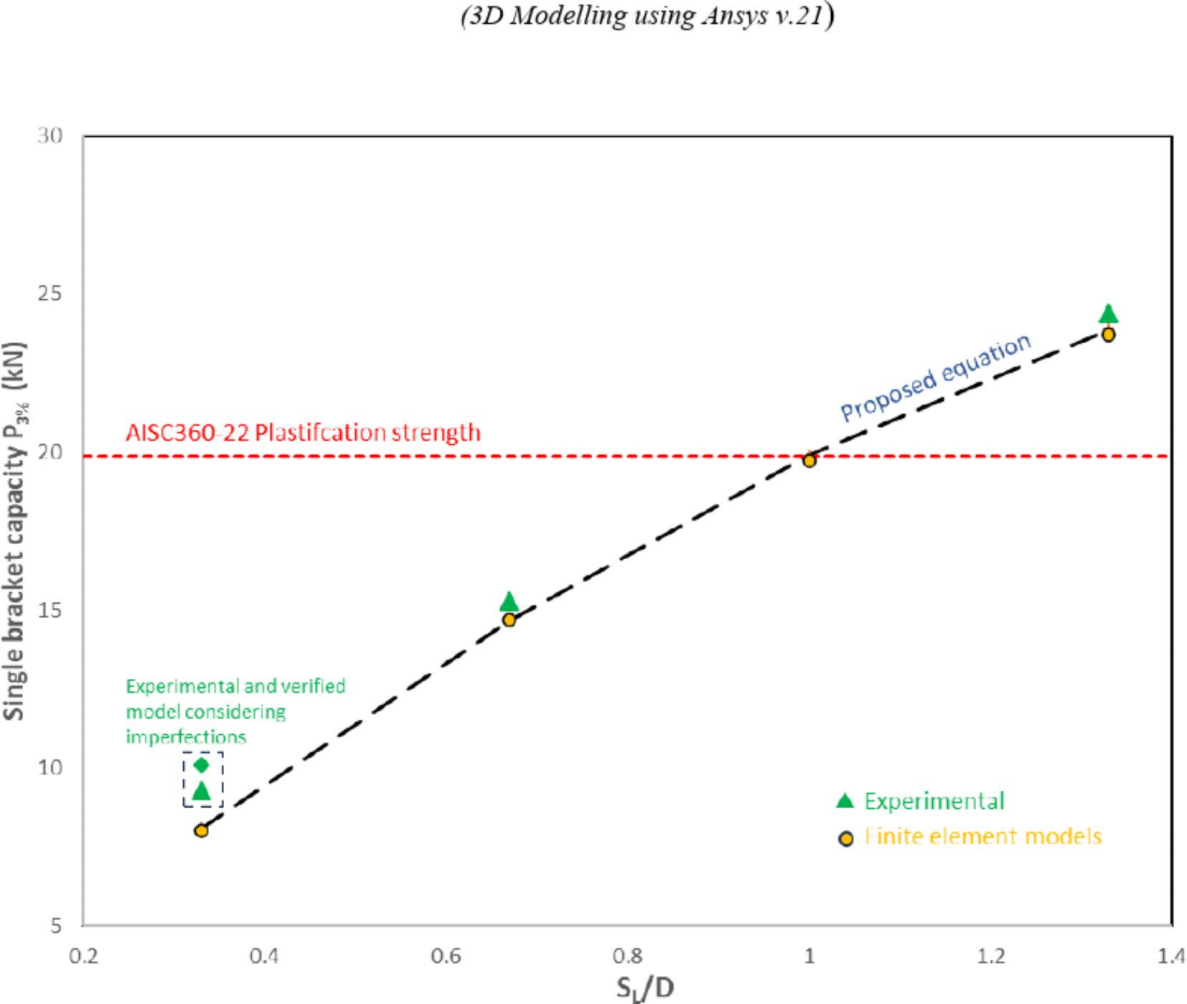
Proposed modification to AISC360-22 equation.

For the studied joint with double brackets, featuring a specific configuration with a transverse spacing of 215 mm, and a force eccentricity of -18 mm, the internal angle between the brackets is 112 degrees. The bracket width is 75 mm, and the chord diameter is 300 mm in all studied specimens, resulting in a constant (Bb/D) ratio of 0.25. International codes list design equations for the X-type branch plates-to-CHS. AISC360-22^15^ and Design Guide 24 provide the same general equation for chord plastification of T- and X-type joints. Table (7) and Fig. 25 indicate that the AISC-22 equation provides results closely matching those of joints with longitudinal spacing equal to the CHS diameter for this particular internal angle (112°). This equation is derived from the geometry of the connection and the material properties involved. The equation is limited to D/t < 40 and B_b_/D ranging from 0.2 to 1 for X-type connections. These limits are defined based on tests conducted to date. this equation is formulated as following:

$${R}_{n}Sin\theta ={F}_{y}{t}^{2}\left(\frac{5.5}{1-0.81\frac{{B}_{b}}{D}}\right){Q}_{f}$$

where θ represents the plate inclination angle.

Q_f_ represents the chord-stress interaction parameter.

Although the D/t ratio for tested specimens exceeds the specified limit of 40, this equation provided results closely matching those of joints with longitudinal spacing equal to the CHS diameter, as presented in Table 7. The increased joints strength, attributed to the smaller internal angle between brackets, offset the strength reduction caused by stress interference from bracket replication.

**Table 7:** Results of AISC360-22 equation vs experimental and FE models.

Specimen ID	P1_3%EXP._ (kN)	P1_3%FE_ (kN)	t (mm)	F_y_ (Mpa)	P1,_EQN_ (kN)	P_1,AISC_/P1_3%EXp_	P_1,AISC_/P1_3%FE_
S-100–300-1	-	19.72	3	320	19.86	-	1.01
S-75–300-1	25.00	29.48	4	255	28.13	1.12	0.95
S-60–300-1	33.30	39.11	5	245	42.24	1.26	1.08
S-100–300-3*	9.30	10.10	3	320	19.86	2.13	1.97
S-100–400-2	15.30	14.67	3	320	19.86	1.30	1.35
S-100–400-1	24.40	23.70	3	320	19.86	0.81	0.84

*Specimen with imperfections.

As the longitudinal spacing between brackets decreases, stress interference increases, leading to a reduction in strength and vice versa. Figure 25 provides the results for the capacity of specimens with different longitudinal spacing values. A quadratic regression analysis was performed to derive a modification factor (A) that can be applied to the AISC-22 equation to account for the effect of various longitudinal spacing values.

The strength of the studied joint for different longitudinal spacing could be expressed as follows:

$${P}_{1}=A\left(\frac{5.5}{1-0.81\frac{{B}_{b}}{D}}\right){F}_{y}{t}^{2}$$

where the modification factor A is defined by the following equation:

$$A=0.018+ 1.272(\text{SL}/\text{D}) -0.288 ($$ S_L_/D)^2^.

The modified equation, incorporating this factor, produces results that align well with data obtained from verified models. The comparisons were conducted after normalizing yield strength across all specimens, as demonstrated in Table 8.

**Table 8:** Proposed simplified equation results vs normalized FE models results.

Specimen ID	P1_3%FE_ (kN)	B_b_/D	S_L_/D	A	P1_,EQN_ (kN)	P_1,EQN_/P1_3%FE_
S-100–300-3*	8.00	0.25	0.33	0.406	8.07	1.008
S-100–400-2	14.67	0.25	0.67	0.740	14.65	0.999
S-100–300-1	19.72	0.25	1	1.002	19.90	1.009
S-100–400-1	23.70	0.25	1.33	1.200	23.84	1.006
S-75–300-1**	34.00	0.25	1	1.002	35.35	1.039
S-60–300-1**	51.42	0.25	1	1.002	55.28	1.075

*Normalized specimen with no imperfections used for comparison.

**Normalized specimens with F_y_ = 320 MPa used for comparison.

Testing the application of the modification factor “A” is needed to derive a more general strength equation for the case of double bracket-to-CHS joints.

## Conclusions and observations

This paper presents a robust methodology for testing double bracket-to-CHS joints subjected to double tensile forces with opposing directions, employing both experimental and numerical approaches. These joints are mainly supporting tensile membrane fabric. Eight joints are experimentally tested. Finite element modeling technique is used to simulate the tested specimens. The accuracy of the FE results is validated against the experimental results. The following conclusions are drawn.

- Increasing the diameter-to-thickness (D/t) ratio leads to a significant reduction in joint strength. The joint strength is increased by 160% for D/t = 60 compared to D/t = 100.
- Specimens with larger longitudinal spacing (S_L_ = 1.33D) is 26% lower than specimens with a smaller longitudinal spacing (S_L_ = 0.33D). However, the single load capacity was increased by 96% for the same specimens.
- The behavior of joint undergoes a transformative shift with alterations in joint eccentricity. For the joint with positive eccentricity, where the forces line of action is located outside the circular cross section, lower joint strength is observed for this case and sudden punching shear occurred.
- The incorporation of a transverse stiffener demonstrates notable effectiveness in increasing the joint strength by 25% and preventing bracket tip fracture until complete flattening occurs.
- A simplified design strength equation is introduced for the double bracket-to-CHS members. The equation accounts for the longitudinal spacing between brackets.

## Data availability statement

The authors confirm that all data used to support the findings are included within the article. Raw data of this study is available from the corresponding author, upon a reasonable request.

## Abbreviations

AISC: American Institute of Steel Construction
CIDECT: Comité International pour le Développement et l’Etude de la Construction Tubulaire
CHS: Circular hollow section
RHS: Rectangular hollow section
FE: Finite element
Fy: Steel material yield strength
Fu: Steel material ultimate strength
E: Steel material young’s modulus
D: Diameter of circular hollow section
t (t_o_): Thickness of circular hollow section
B_b_: Branch bracket width
t_p_: Branch bracket thickness
S_L_: Bracket longitudinal spacing
S_t_: Bracket transverse spacing
e: Force line of action eccentricity (+ ve for line of action outside CHS perimeter)
P1_3%_: Single bracket horizontal load corresponding to 3%D deformation limit
P1_f_: Single bracket horizontal load at fracture point or punching shear
δ_1_: Joint crown vertical deformation at point (1) (+ ve for deformations inside CHS perimeter)

## References

[1] Lu, L.H., de Winkel, G.D., Yu, Y. and Wardenier, J. ,Deformation limit for the ultimate strength of hollow section joints., Proceedings of the 6th International Symposium on Tubular Structures. Melbourne, Australia, A.A. Balkema, (1994) 341–347.

[2] J. Wardenier, Y. Kurobane, J.A. Packer, G.J. van der Vegte, X.-L. Zhao, Design Guide for Circular Hollow Section (CHS) Joints under Predominantly Static Loading, 2nd ed., CIDECT, Geneva, Switzerland, (2008).

[3] International Institute of Welding (2009). Static design procedure for welded hollow section joints: Recommendations, 3rd Edition. IIW Doc. XV-1329–09. IIW Annual Assembly, Singapore.

[4] Voth, A. P. & Packer, J. A. Branch plate-to-circular hollow structural section connections:experimental investigation and finite element modeling. *J. Struct. Eng.* **138**(8), 995–1006 (2011).

[5] Voth, A. P. & Packer, J. A. Numerical study and design of T-type branch plate-to circular hollow section connections. *Eng. Struct.* **41**, 477–489 (2012).

[6] M.M. Hassan, H. Ramadan, M. Abdel-Mooty, S.A. Mourad, Experimental and numerical study of one-sided branch plate-to-circular hollow section connections,Steel Compos. Struct., Int. J. 19 (4) (2015) 877–895.

[7] Zapata, L. M., Graciano, C. & Zapata-Medina, D. G. Ultimate strength of transversal T-branch plate-to-CHS connections under compression. *Thin walled structures* **112**, 92–97 (2017).

[8] Nassiraei, H., Zhu, L., Lotfollahi-Yaghin, M. A. & Ahmadi, H. Static capacity of tubular X-joints reinforced with collar plate subjected to brace compression. *Thin-Walled Structures* **119**, 256–265 (2017).

[9] Garifullin, M. et al. Finite element model for rectangular hollow section T joints. *Rakenteiden Mekaniikka (Journal of Structural Mechanics)* **51**(3), 15–40 (2018).

[10] Nassiraei, H. Static strength of tubular T/Y-joints reinforced with collar plates at fire induced elevated temperature. *Marine Structures* **67**, 102635 (2019).

[11] Lan, X., Chan, T. M. & Young, B. Experimental study on the behaviour and strength of high strength steel CHS T-and X-joints. *Engineering Structures* **206**, 110182 (2020).

[12] Nassiraei, H. Local joint flexibility of CHS T/Y-connections strengthened with collar plate under in-plane bending load: parametric study of geometrical effects and design formulation. *Ocean Engineering* **202**, 107054 (2020).

[13] Lan, X., Chan, T. M. & Young, B. Structural behaviour and design of high strength steel CHS T-joints. *Thin-Walled Structures* **159**, 107215 (2021).

[14] Kožich, M., Jehlička, P., Kuříková, M., Wald, F., Bu, X. D., Packer, J. A., & Kabeláč, J. (2021). Strain Design Limit for Hollow Section Joints. *ce/papers*, *4*(2–4), 2488–2494.

[15] American Institute of Steel Construction (2022). Specification for structural steel buildings. ANSI/AISC 360–22. American Institute of Steel Construction, Chicago, USA.

[16] Zuo, W. et al. Experimental investigation on compressive behavior of corroded thin-walled CHS T-joints with grout-filled GFRP tube repairing. *Thin-Walled Structures* **175**, 109222 (2022).

[17] Chen, M. T., Zuo, W. & Young, B. Tests of cold-formed steel T-joints with semi-oval hollow section chord. *Journal of Structural Engineering* **149**(8), 04023099 (2023).

[18] Nassiraei, H. Probability distribution models for the ultimate strength of tubular T/Y-joints reinforced with collar plates at room and different fire conditions. *Ocean Engineering* **270**, 113557 (2023).

[19] Fayed, A., Hammad, A. & Shaat, A. Optimal location of a single through-bolt for efficient strengthening of CHS K-joints. *Structural Engineering and Mechanics* **89**(1), 61–75 (2024).

[20] AISC Design Guide 24: Hollow Structural Section Connections, (2024).

[21] Ansys Help, https://ansyshelp.ansys.com/

